# Metabolic rewiring induced by ranolazine improves melanoma responses to targeted therapy and immunotherapy

**DOI:** 10.1038/s42255-023-00861-4

**Published:** 2023-08-10

**Authors:** Marta Redondo-Muñoz, Francisco Javier Rodriguez-Baena, Paula Aldaz, Adriá Caballé-Mestres, Verónica Moncho-Amor, Maddalen Otaegi-Ugartemendia, Estefania Carrasco-Garcia, Ana Olias-Arjona, Irene Lasheras-Otero, Eva Santamaria, Ana Bocanegra, Luisa Chocarro, Abby Grier, Monika Dzieciatkowska M, Claudia Bigas, Josefina Martin, Uxue Urdiroz-Urricelqui, Florencio Marzo, Enrique Santamaria, Grazyna Kochan, David Escors, Ignacio Marcos Larrayoz, Holger Heyn, Angelo D’Alessandro, Camille Stephan-Otto Attolini, Ander Matheu, Claudia Wellbrock, Salvador Aznar Benitah, Berta Sanchez-Laorden, Imanol Arozarena

**Affiliations:** 1 Cancer Signaling Unit, Navarrabiomed, Hospital Universitario de Navarra (HUN), Universidad Pública de Navarra (UPNA), Pamplona, Spain; 2 Health Research Institute of Navarre (IdiSNA), Pamplona, Spain; 3 Instituto de Neurociencias, CSIC-UMH, Sant Joan d’Alacant, Spain; 4 Institute for Research in Biomedicine (IRB Barcelona), The Barcelona Institute of Science and Technology (BIST), Barcelona, Spain; 5 Cellular Oncology Group, Biodonostia Health Research Institute, San Sebastian, Spain; 6 CIBER de Fragilidad y Envejecimiento Saludable (CIBERfes), Madrid, Spain; 7 Hepatology Program, CIMA, CCUN, University of Navarra, Pamplona, Spain; 8 CIBERehd, Instituto de Salud Carlos III, Madrid, Spain; 9 Oncoimmunology Group, Navarrabiomed, Hospital Universitario de Navarra (HUN), Universidad Pública de Navarra (UPNA), Pamplona, Spain; 10 Department of Biochemistry and Molecular Genetics, University of Colorado Anschutz Medical Campus, Aurora, CO USA; 11 Clinical Neuroproteomics Unit, Navarrabiomed, Hospital Universitario de Navarra (HUN), Universidad Pública de Navarra (UPNA), Pamplona, Spain; 12 Biomarkers and Molecular Signaling Group, Center for Biomedical Research of La Rioja (CIBIR), Foundation Rioja Salud, Logroño, Spain; 13 Unidad Predepartamental de Enfermería, Universidad de La Rioja (UR), Logroño, Spain; 14 CNAG-CRG, Centre for Genomic Regulation (CRG), Barcelona Institute of Science and Technology (BIST), Barcelona, Spain; 15 IKERBASQUE, Basque Foundation for Science, Bilbao, Spain; 16 Department of Health Sciences, Universidad Pública de Navarra (UPNA), Pamplona, Spain; 17 Catalan Institution for Research and Advanced Studies (ICREA), Barcelona, Spain

**Keywords:** Cancer therapeutic resistance, Melanoma

## Abstract

Resistance of melanoma to targeted therapy and immunotherapy is linked to metabolic rewiring. Here, we show that increased fatty acid oxidation (FAO) during prolonged BRAF inhibitor (BRAFi) treatment contributes to acquired therapy resistance in mice. Targeting FAO using the US Food and Drug Administration-approved and European Medicines Agency-approved anti-anginal drug ranolazine (RANO) delays tumour recurrence with acquired BRAFi resistance. Single-cell RNA-sequencing analysis reveals that RANO diminishes the abundance of the therapy-resistant NGFR^hi^ neural crest stem cell subpopulation. Moreover, by rewiring the methionine salvage pathway, RANO enhances melanoma immunogenicity through increased antigen presentation and interferon signalling. Combination of RANO with anti-PD-L1 antibodies strongly improves survival by increasing antitumour immune responses. Altogether, we show that RANO increases the efficacy of targeted melanoma therapy through its effects on FAO and the methionine salvage pathway. Importantly, our study suggests that RANO could sensitize BRAFi-resistant tumours to immunotherapy. Since RANO has very mild side-effects, it might constitute a therapeutic option to improve the two main strategies currently used to treat metastatic melanoma.

## Abstract (web summary)

In this study, the anti-anginal drug ranolazine is found to sensitize BRAF inhibitor-resistant melanoma to targeted therapy and immunotherapy by rewiring fatty acid oxidation and the methionine salvage pathway.

## Main

Cutaneous melanoma is the most lethal skin cancer. Novel targeted therapies for individuals with BRAF mutations and immune checkpoint inhibitors have significantly improved the clinical outcome of melanoma^1,2^. Nevertheless, the effectiveness of these treatments is challenged by limitations in sustained responses and relapse of drug-resistant disease^3^.

Resistance to inhibitors of BRAF (BRAFi) and MEK (MEKi) is linked to non-mutational adaptation of melanoma cells during the early response and the following drug-tolerant phase of treatment^4^. Therefore, melanoma cells rewire cellular signalling and establish transcriptional programmes that provide a survival advantage in the presence of these MAPK inhibitors (MAPKi)^5^. For instance, one such non-mutational event involves the upregulation of the melanocyte lineage-specific transcription factor MITF^4,6,7^. In a mutually exclusive manner, another population of melanoma cells upregulates a transcriptional programme linked to the receptor tyrosine kinase AXL^8^. Thus, during treatment, the response of individual melanoma cells can display great plasticity, and intra-tumour heterogeneity can be observed with regard to multiple co-occurring transcriptional states^5^. Apart from the ‘MITF activity’-driven state, an AXL high ‘invasive’ and a ‘neural crest stem cell’ (NCSC) state, characterized by NGFR expression, also arise following MAPKi treatment^6^. These different melanoma cell states are relevant for the outcome of treatment, because they can individually establish MAPKi-resistant tumours with distinct characteristics. For instance, melanomas primarily composed of the NGFR-expressing NCSC state are the most refractory to not only targeted therapy, but also immunotherapy^6,9^.

Another transcriptional state peaking early during the treatment with MAPKi is linked to nutrient starvation^6^. The occurrence of this ‘starved-like melanoma cell’ state emphasizes that metabolic rewiring represents a major event during MAPKi treatment^10^. Already the early response to BRAF inhibition involves a profound reduction in glucose uptake and glycolysis^6,11,12^, while oxidative phosphorylation and the expression of a mitochondrial biogenesis programme are upregulated^13,14^. While glycolysis is inhibited, melanoma cells can use CPT1A to drive mitochondrial FAO to maintain their viability^11^. Short-term MAPKi treatment can also select for a subpopulation of cells that uses peroxisomal FAO to tolerate MAPK inhibition^15^. Thus, FAO appears to be important for survival during the early response and drug-tolerant phase of targeted therapy, which is also reflected in the upregulation of the fatty acid transporter CD36 during this phase^6,11^. Nevertheless, once MAPKi resistance is established, the metabolic network has again rewired. In melanomas from progressed patients and in acquired resistant melanoma cells, glycolysis is frequently re-established^12^, and glutamine dependence is observed in selected resistant cell populations^16,17^. However, the role of FAO in the state of acquired resistance to MAPKi is unknown.

Besides its role in the early response to MAPKi inhibition, FAO also contributes to age-dependent resistance to MAPKi, whereby FAO in melanoma cells is fuelled by aged dermal fibroblasts, known to acquire adipocyte traits favouring a lipid secretome^6,18^. We have shown that activation of FAO in tumour circulating cells contributes to melanoma progression^19^ and emerging evidence implies that FAO plays an important role in the immune tumour microenvironment^20^. Considering this complexity, we aimed to dissect the role of FAO and its inhibition in the context of therapy resistance in melanoma.

## Results

### Fatty acid beta-oxidation increases during resistance acquirement

**Fig. 1:**
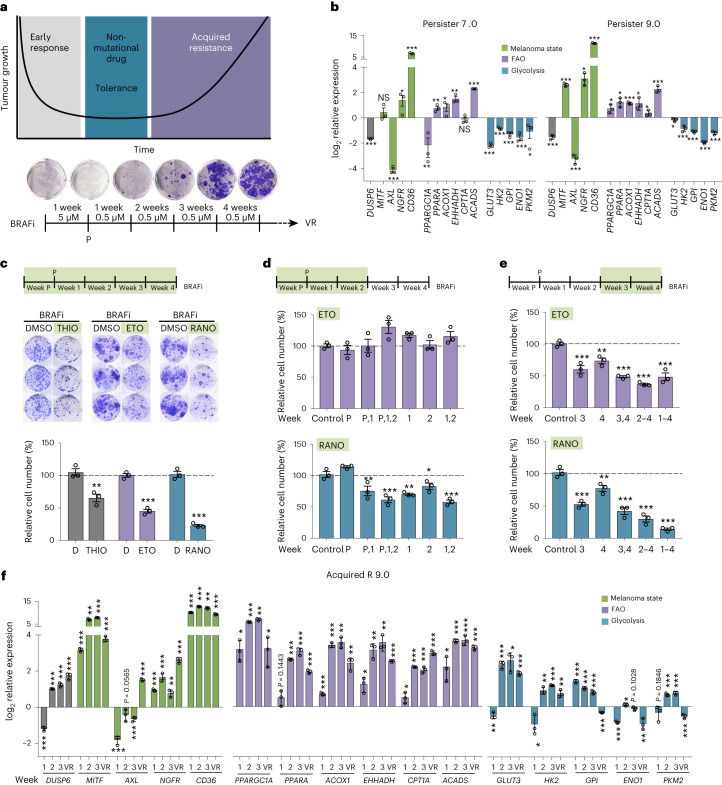
Fatty acid oxidation is upregulated during BRAFi-acquired resistance development. **a**, Protocol for monitoring the establishment of vemurafenib (BRAFi) resistance. P, persister; VR, vemurafenib resistant. **b**, Quantitative PCR (qPCR) analysis of the indicated genes in BRAFi persister cells. Shown are expression data from persister cells from two independent acquired resistance experiments (7.0 and 9.0) relative to untreated cells. Data are presented as the mean ± s.d. based on *n* = 3 sample replicates analysed by two-tailed unpaired *t*-test. *P < 0.05; **P < 0.01; ***P < 0.001. **c**, Colony formation assay (CFA) quantification for A375 cells treated with BRAFi in the absence (dimethylsulfoxide (DMSO)) or presence of 2 µM THIO, 100 µM ETO or 100 µM RANO added once per week. Data are presented as the mean ± s.e.m. based on *n* = 3 biological replicates analysed by two-tailed unpaired *t*-test. **P < 0.01; ***P < 0.001. **d**,**e**, CFA quantification of A375 cells treated with BRAFi in the absence (control) or presence of ETO or RANO added once per week during the early (**d**) or late (**e**) stage of treatment as indicated. Data are presented as the mean ± s.e.m. based on *n* = 3 biological replicates analysed by one-way analysis of variance (ANOVA) with Dunnett’s multiple-comparisons test. *P < 0.05; **P < 0.01; ***P < 0.001. **f**, qPCR analysis of the indicated genes in A375 cells treated with BRAFi. Shown is the expression at weeks 1, 2 and 3 after the establishment of persister 9.0 and in BRAFi-resistant VR 9 cells relative to untreated cells. Data are presented as the mean ± s.d. based on *n* = 3 sample replicates analysed by two-tailed unpaired *t*-test. *P < 0.05; **P < 0.01; ***P < 0.001. NS, not significant. Source data

**Extended Data Fig. 1:**
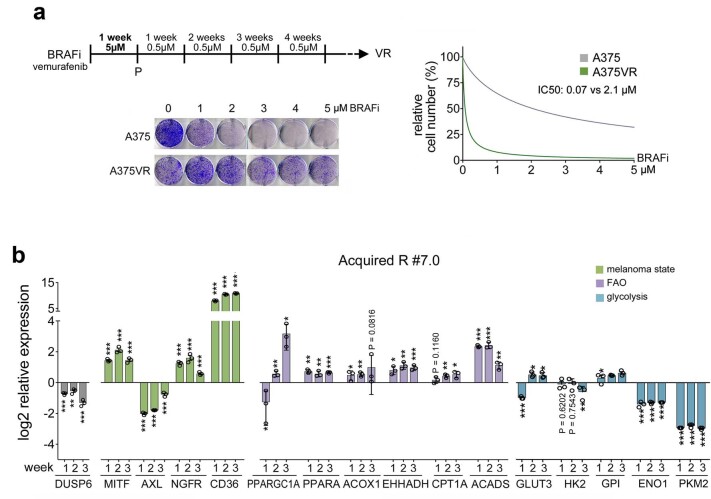
Elevated FAO regulator expression during BRAFi-acquired resistance development. **a**, Dose response curve for vemurafenib (BRAFi) in A375 cells and A375VR cells. **b**, qPCR analysis of the indicated genes in A375 cells treated with BRAFi. Shown is the expression at week 1, 2 and 3 after the establishment of persister #7.0. Data are presented as the mean ± s.d. based on *n* = 3 sample replicates analysed by two-tailed unpaired *t*-test. *P < 0.05; **P < 0.01; ***P < 0.001. Source data

We wanted to examine the effect of BRAFi on metabolic rewiring not only in cells that survived the initial drug insult, but also throughout the course of acquired resistance development. To this end, human BRAF^V600E^ A375 melanoma cells were treated with a high dose of vemurafenib (BRAFi) for 7 d, during which time only ‘persister’ cells survived (Fig. 1a). These persister cells were then treated with a lower concentration of BRAFi for 4 weeks at which point vemurafenib-resistant populations had been established, which maintained their acquired resistance when further propagated as A375VR cells (Fig. 1a and Extended Data Fig. 1a).

Analysis of persister cells in our model confirmed effective inhibition of BRAF, indicated by the reduction in *DUSP6* expression, as well as changes in markers linked to drug-tolerant transcriptional states (MITF, AXL, NGFR and CD36)^6^, the downregulation of key glycolysis regulators^12^ and upregulation of peroxisomal and mitochondrial FAO markers^11,15^ (Fig. 1b). Individual changes in gene expression, particularly regarding cell-state markers displayed some variability among different experiments (Fig. 1b and not shown), reflecting the result of plasticity, which can be observed even within a single MAPKi-treated melanoma cell line^21,22^.

To assess whether FAO was relevant for acquiring resistance to BRAFi, we included different FAO inhibitors in our treatment protocol. A375 cells were treated with BRAFi as previously described in the presence or absence of either thioridazine (THIO), which targets peroxisomal beta-oxidation^23^, etomoxir (ETO), an inhibitor of the mitochondrial fatty acid transporter CPT1A^24^ and RANO, which blocks acetyl-CoA production from fatty acids in mitochondria^25^. All three inhibitors significantly reduced the establishment of vemurafenib-resistant A375 colonies (Fig. 1c).

Because FAO has previously been linked to the initial drug-tolerant phase of BRAFi^11,15^, we expected that inhibition in the early phase of our treatment protocol would be most effective. To address this point, we treated cells once weekly either during the early phase or during the late phase of our treatment protocol. We focused on RANO, because it is a well-tolerated US Food and Drug Administration (FDA)-approved drug with translational potential, and we applied ETO as a control, because it has been previously used in the context of early MAPKi adaptation^11^.

In line with the previously reported finding that CPT1A inhibition in melanoma cells reactivates MAPKi-suppressed glycolysis and counteracts the BRAFi growth inhibitory effect^11^, ETO was ineffective when added early on during the treatment (Fig. 1d). However, RANO profoundly reduced the establishment of acquired resistance colonies (Fig. 1d). Intriguingly, RANO was still effective when administered 2 weeks after the occurrence of persister cells (Fig. 1e), suggesting that FAO is also involved in melanoma cell propagation later in treatment during the establishment of acquired resistance. This is supported by the fact that ETO also inhibited the establishment of BRAFi-resistant colonies when applied later (Fig. 1e). It appears that a potential effect of ETO on glycolysis loses its relevance in later stages of resistance development.

Our results thus far suggested that FAO is enhanced throughout the whole BRAFi treatment course. To test this idea, we monitored the expression of the respective marker genes throughout the development of acquired resistance in our model and found that the upregulation of FAO regulators was maintained throughout treatment (Fig. 1f and Extended Data Fig. 1b). On the other hand, in line with previous findings^12^, the downregulation of some glycolysis regulators was reversed as early as 2 weeks after the generation of persister cells (Fig. 1f). However, while the recovery of glycolysis marker expression showed distinct heterogeneity in separate acquired resistance experiments, the expression of FAO regulators during the establishment of acquired resistance was consistently detectable (Fig.1f and Extended Data Fig. 1b). Notably, several glycolysis regulators displayed reduced expression in resistant cells, but FAO regulator expression was still enhanced (Fig. 1f), suggesting increased lipid metabolism in BRAFi-acquired resistant A375VR cells.

### Ranolazine moderates the BRAFi-lipidome and mitochondrial respiration

**Fig. 2:**
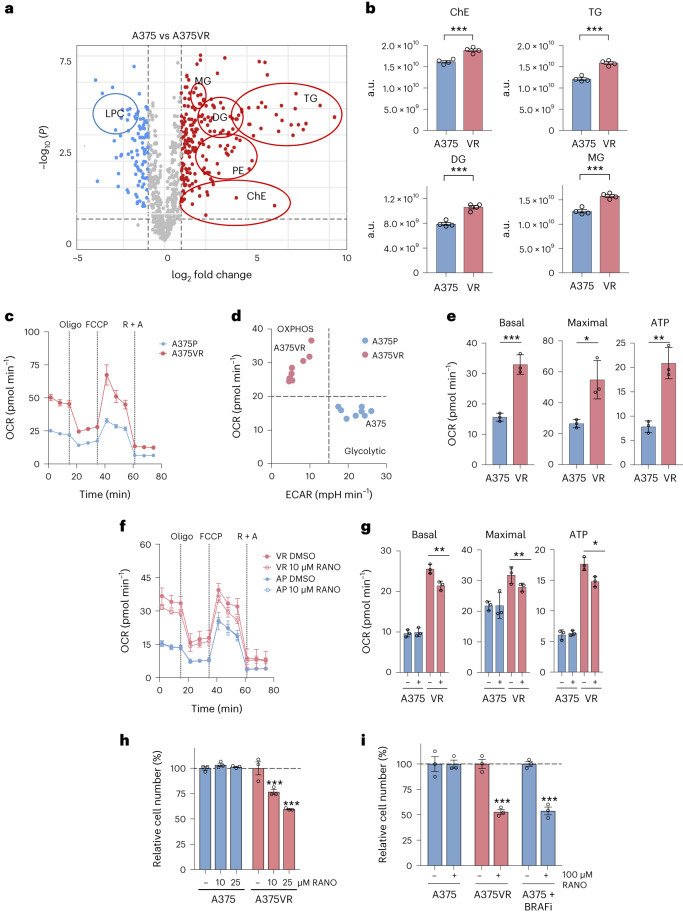
Ranolazine reduces mitochondrial respiration in BRAFi-resistant cells. **a**, Volcano plot showing the log_2_ fold change of lipids in A375 versus A375VR cells (LPC: lysophosphatidylcholine; MG, monoacylglycerols; DG, diacylglycerols; TG, triacylglycerols; PE, phosphatidylethanolamines and ChE, cholesterol esters). **b**, Total levels (integrated peak areas; a.u., arbitrary units) of lipids divided by classes in A375 versus A375VR cells. Data are presented as the mean ± s.e.m. based on *n* = 4 biological replicates analysed by two-tailed unpaired *t*-test. ***P < 0.001. **c**, Representative oxygen consumption rate (OCR) of A375 and A375VR cells. Oligo, oligomycin; FCCP, carbonyl cyanide-p-trifluoromethoxyphenylhdrazone; R, rotenone; A, antimycin A. Data are shown as the mean ± s.d. with *n* = 3 replicate wells. **d**, Basal OCR of A375VR and A375 cells was plotted against their basal extracellular acidification rate (ECAR). Data are from *n* = 3 biological replicates of three replicate wells. **e**, Basal or maximal respiration and ATP production in A375 and A375VR cells. Data are presented as the mean ± s.d. based on *n* = 3 replicate wells analysed by two-tailed unpaired *t*-test. **P < 0.01; ***P < 0.001. **f**,**g** Representative OCR (**f**) and basal or maximal respiration and ATP production (**g**) of A375 and A375VR cells in the absence (DMSO) or presence of 10 µM RANO. Data are shown as the mean ± s.d. based on *n* = 3 replicate wells and analysed by one-way ANOVA with uncorrected Fisher’s least significant difference (LSD). *P < 0.05; **P < 0.01. **h**,**i** Relative cell number of A375 or A375VR cells (**h**) treated daily with 10 µM or 25 µM RANO or treated once per week with 100 µM RANO with or without 0.5 µM BRAFi (**i**) as indicated. Control cells were treated with DMSO. Data are presented as the mean ± s.e.m. based on *n* = 3 biological replicates and analysed by one-way ANOVA with Sidak’s multiple-comparisons test. ***P < 0.001. Source data

**Extended Data Fig. 2:**
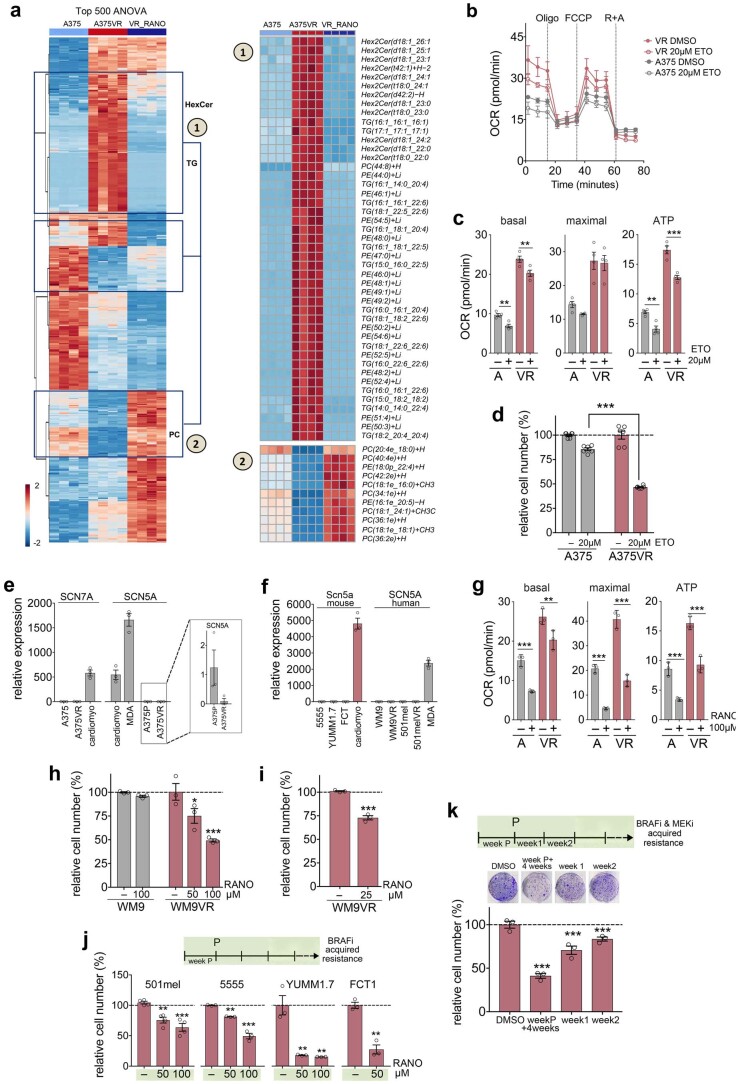
Ranolazine reverses BRAFi induced changes in the lipidome and counteracts BRAFi acquired resistance. **a**, Heatmap of the top 500 significant (FDR corrected, ANOVA) lipids impacted by VR or VR_RANO in A375 cells; two main clusters were identified showing either increases or decreases in response to BRAFi treatment, respectively (normalized by VR_RANO). In the VR group, Hex2Ceramides, triacylglycerols (TGs) and phosphatidylethanolamines (PE) were over-represented, while in the VR_RANO group phosphatidylcholines (PCs) were the most common lipid class increasing after reduction by BRAFi treatment. **b**,**c** (**b**) OCR and (**c**) basal or maximal respiration and ATP production of A375 and A375VR cells in the absence (DMSO) or presence of 20 µM ETO. For (**b**) data are shown as mean ± s.d. with *n* = 2 replicate wells. For (**c**) data are presented as mean ± s.em. based on *n* = 2 biological replicates performed as *n* = 2 replicate wells and analysed by one-way ANOVA with uncorrected Fisher’s LSD. **P < 0.01; ***P < 0.001. **d**, Relative cell number of A375 or A375VR cells treated daily with 20 µM ETO. DMSO was set as 100%. Data are presented as mean ± s.em. based on *n* = 3 biological replicates performed as *n* = 2 replicate wells and analysed by one-way ANOVA with uncorrected Fisher’s LSD. ***P < 0.001. **e**,**f** qPCR analysis of *SCN5A/Scn5a* and *SCN7A* expression in (**e**) A375 and A375VR cells and (**f**) in the indicated human or mouse melanoma cell lines. Mouse or human cardiomyocytes and MDA-MB-231 cells served as positive controls. Data are presented as mean ± s.e.m based on *n* = 3 biological replicates. **g**, basal or maximal respiration and ATP production of A375 and A375VR cells in the absence or presence of 100 µM RANO. Data are presented as mean ± s.d. based on *n* = 3 replicate wells and analysed by one-way ANOVA with uncorrected Fisher’s LSD. **P < 0.01; ***P < 0.001. **h**, CFA quantification of WM9 or WM9VR cells treated with 50 µM or 100 µM RANO once per week. Data are presented as mean ± s.em. based on *n* = 3 biological replicates, and analysed by one-way ANOVA with Sidak’s multiple comparison test. *P < 0.05; ***P < 0.001. (**i**) CFA quantification of WM9VR cells treated daily with 25 µM RANO. Data are presented as mean ± s.em. based on *n* = 3 biological replicates analysed by two-tailed unpaired *t*-test. ***P < 0.001. **j**, 501mel, 5555, YUMM1.7 and FCT1 cells were treated with high concentrations of BRAFi (10 µM for 501mel and 5555; 5 µM for YUMM1.7, FCT1) for 1 week and then treatment continued at a lower concentration of BRAFi (1 or 0.5 µM, respectively) until acquired resistance was established. BRAFi treatment was in the absence or presence of 50 or 100 µM RANO added once per week. Data are presented as mean ± s.em. based on *n* = 4 biological replicates for 501mel and *n* = 3 biological replicates for 5555, YUMM1.7 and FCT1, and analysed by one-way ANOVA with Sidak’s multiple comparison test or two-tailed unpaired *t*-test. **P < 0.01; ***P < 0.001. **k**, A375 cells were treated with high concentrations of BRAFi and MEKi (5 µM and 100 nM, respectively) for 1 week and then treatment continued at a lower concentration (0,5 µM and 10 nM, respectively) until acquired resistance was established. MAPK inhibitor treatment was in the absence or presence of 100 µM RANO added once per week. Data are presented as mean ± s.em. based on *n* = 3 biological, and analysed by one-way ANOVA with Dunnett’s multiple comparisons test. ***P < 0.001. Source data

Lipidomics analysis of A375VR cells compared to naïve A375 cells confirmed altered lipid metabolism with an increase in several classes of fatty acids and lipids (Fig. 2a,b and Extended Data Fig. 2a) comparable to what has been observed previously in BRAFi persister cells that had switched to FAO^15^. In line with this, oxygen consumption was increased in A375VR cells compared to naïve A375 cells (Fig. 2c), and while A375 cells displayed a glycolytic phenotype, the bioenergetic profile of A375VR cells had switched to increased mitochondrial activity (Fig. 2d,e). Targeting CPT1A with ETO resulted in reduced mitochondrial ATP production in A375VR and A375 cells (Extended Data Fig. 2b,c). However, while this only slightly impacted on the growth of A375 cells, the growth of A375VR cells was strongly reduced (Extended Data Fig. 2d).

Although the specific target of RANO in fatty acid metabolism is unknown^26,27^, it reversed a major part of the BRAFi-induced changes in the A375VR lipidome (Extended Data Fig. 2a). In cardiac myocytes RANO selectively blocks late sodium current by acting on the voltage-gated sodium channel Nav1.5 (SCNA5)^26^. However, melanoma cells express negligible amounts of SCNA5 or SCN7A, another cardiac channel isoform (Extended Data Fig. 2e,f).

Furthermore, 10 µM RANO, a dose that can effectively inhibit late sodium current in cardiac myocytes^28^, has no effect on oxygen consumption in parental A375 cells (Fig. 2f,g). In contrast, in A375VR cells, which express even ten times less SCNA5 than A375 cells (Extended Data Fig. 2e), 10 µM RANO significantly reduced mitochondrial respiration and ATP production (Fig. 2f,g). Thus, although the specific target for RANO in the context of FAO is not known, it selectively reduces mitochondrial respiration in cells in which BRAF inhibition triggers increased FAO.

When added daily, 10 µM RANO also significantly reduced A375VR cell propagation, but did not affect parental A375 cells, and a more potent effect was seen with 25 µM RANO (Fig. 2h). Moreover, the specificity of RANO for A375VR cells over parental A375 cells was maintained in the presence of 100 µM RANO (Fig. 2i), when added once per week as applied in our initial acquired resistance experiments (Fig. 1c–e). This specificity was seen despite 100 µM RANO reducing mitochondrial oxygen consumption not only in A375VR cells but also in parental cells (Extended Data Fig. 2g), a situation similar to what we had observed with ETO (Extended Data Fig. 2b–d). Overall, this suggests that energy derived from oxidative phosphorylation contributes less to the growth of glycolytic A375 cells. However, addition of BRAFi, which suppresses glycolysis, enhanced the sensitivity of A375 cells to RANO (Fig. 2i).

The selectivity of RANO for BRAFi-resistant cells over naïve melanoma cells, as well as its ability to suppress the establishment of BRAFi-acquired resistance was also seen in other human and mouse melanoma cells lines (Extended Data Fig. 2h–j). Moreover, RANO also counteracted the establishment of BRAFi/MEKi-resistant cells, whereby it was more potent when added from the time of the establishment of persister cells (Extended Data Fig. 2k).

### Ranolazine delays the onset of BRAFi-acquired resistance in vivo

**Fig. 3:**
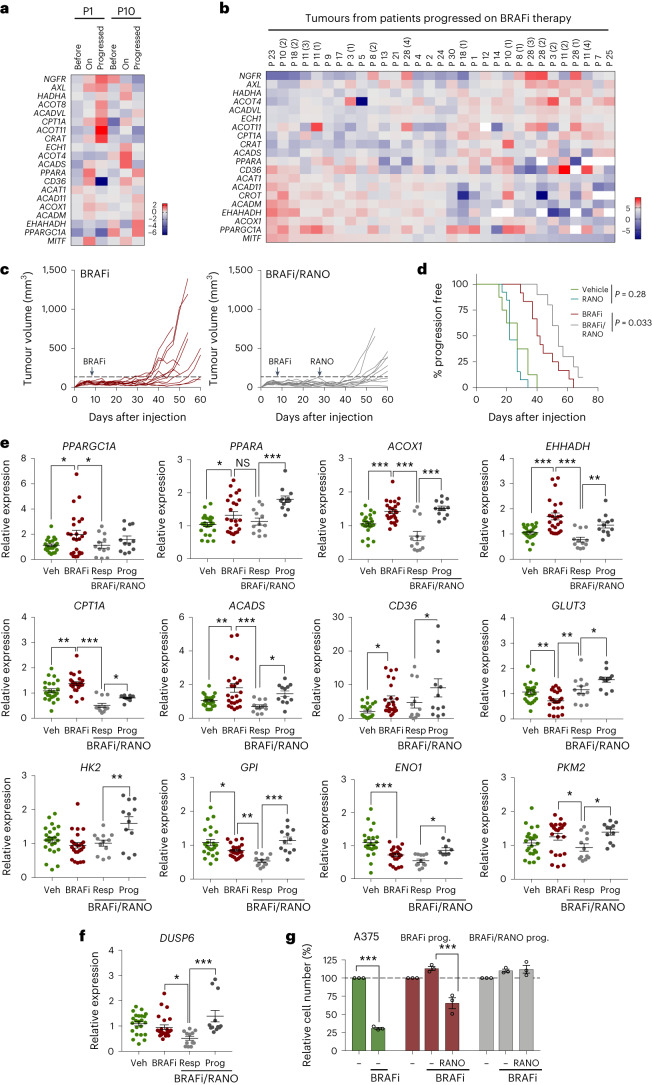
Ranolazine delays BRAFi-acquired resistance in vivo. **a**,**b**, Analysis of publicly available gene expression datasets GSE61992 (ref. ^61^) and GSE50509 (ref. ^62^). Data from 2 patients (GSE61992) before and on treatment, and at the time of progression (**a**) or 31 tumours from 21 patients (GSE50509) before treatment and at the time of progression (**b**) were analysed for expression of the indicated genes. For **b**, the log_2_ fold change at the time of progression relative to ‘before’ is shown. The number of individual tumours from patients with more than one tumour is indicated in brackets. **c**, Growth curves of individual tumours from female mice treated with BRAFi alone (25 mg per kg body weight, daily on day 7) or with BRAFi and RANO (50 mg per kg body weight, daily), which was added at day 28 when BRAFi-treated tumours showed a reduced response to treatment. *n* = 12 tumours in each group. **d**, Kaplan–Meier plots of progression-free survival of female mice treated as indicated. Progression was declared when tumours exceeded a volume of twice the average volume of day 7, when BRAFi treatment commenced (dashed line). Log-rank (Mantel–Cox) test: BRAFi versus BRAFi/RANO hazard ratio (HR) = 2.88. **e**,**f** qPCR analysis of the indicated genes in A375 tumours from female mice treated as indicated. Data are triplicates from *n* = 8 tumours for vehicle or BRAFi, and *n* = 4 tumours for RANO responder (resp) or RANO progressed (prog), respectively, and are presented as the mean ± s.e.m. analysed by one-way ANOVA with uncorrected Fisher’s LSD. *P < 0.05; **P < 0.01; ***P < 0.001. **g**, Relative cell number of cell cultures established from tumours progressed on BRAFi or BRAFi/RANO treated with BRAFi in the absence (DMSO) or presence of RANO. A375 cells served as the control. Data are presented as the mean ± s.e.m. based on *n* = 3 biological replicates analysed by one-way ANOVA with Sidak’s multiple-comparisons test. ***P < 0.001. Source data

**Extended Data Fig. 3:**
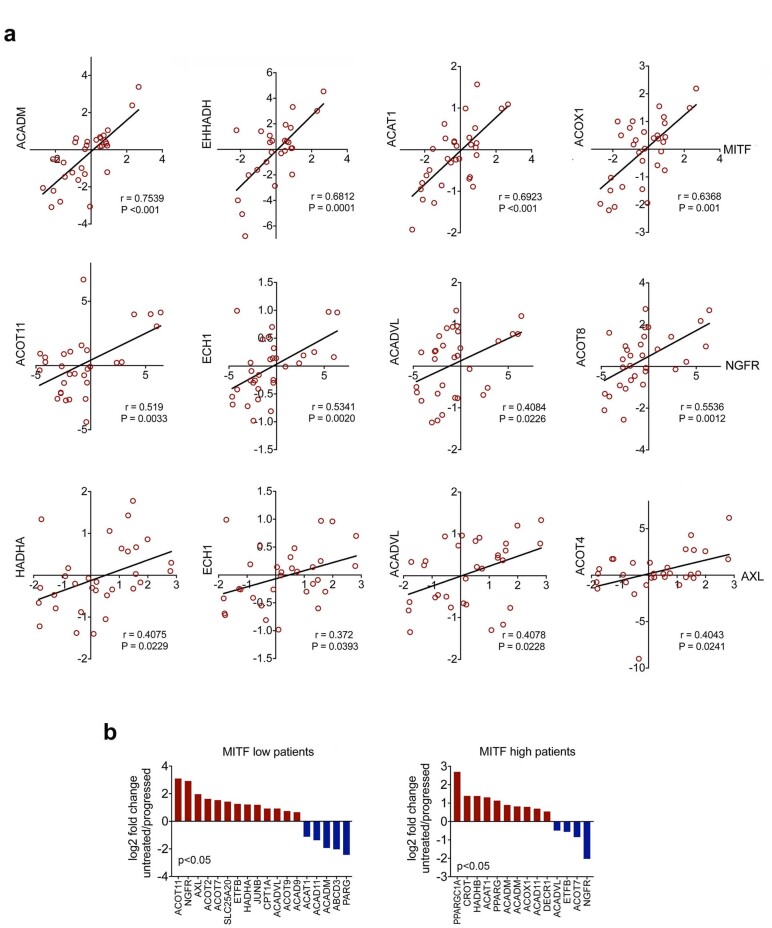
FAO regulator expression in human melanoma tumours. **a**, Pearson correlation of the fold change in expression of the indicated FAO regulators with melanoma state markers AXL, NGFR or MITF respectively in tumours from patients progressed on MAPKi when compared to the same tumours before treatment (data relate to GSE50509). **b**, fold change expression of the indicated genes in groups of patients stratified for high or low MITF expression. Cut-off: *P* < 0.05. Source data

Our data suggest that enhanced FAO occurs as a persistent event during the development of MAPKi resistance. In line with this hypothesis, we found increased expression of FAO-linked genes not only in tumours from individuals in the early stage of treatment (Fig. 3a), but also in tumours of individuals progressed on BRAFi (Fig. 3a,b). Upregulation of mitochondrial as well as peroxisomal FAO genes was seen in all progressed tumours when compared to ‘before’ samples, and, intriguingly, the upregulation of many of these correlated with individual melanoma state markers (Fig. 3b and Extended Data Fig. 3a,b).

**Extended Data Fig. 4:**
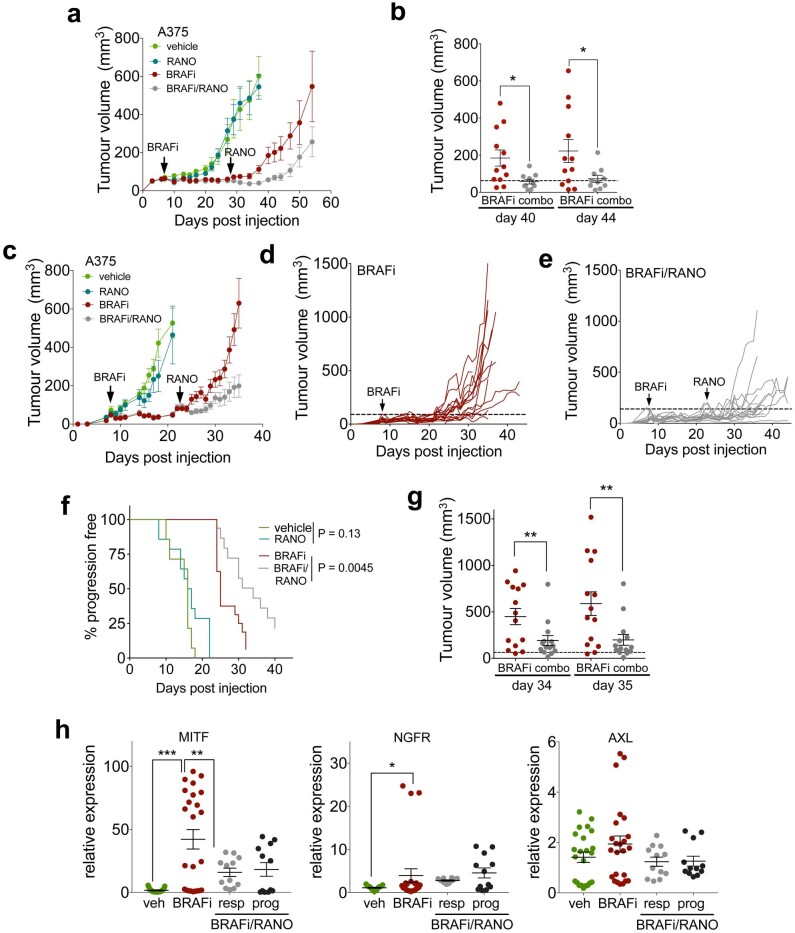
Ranolazine improves BRAFi therapy. **a**, Growth curves of tumours from female mice treated with vehicle, RANO alone 50 mg/kg, daily, BRAFi alone (25 mg/kg, daily) or with BRAFi (25 mg/kg, daily) and RANO (50 mg/kg, daily), RANO was added at day 28 when BRAFi treated tumours showed a reduced response to treatment. Data are presented as mean ± s.e.m. based on *n* = 12 tumours in each group. **b**, Tumour end volume of female mice treated with BRAFi (*n* = 12) or the combination (combo) of BRAFi with RANO (*n* = 10) at days 40 and 44 of treatment. Data are presented as mean ± s.e.m. analysed by one-way ANOVA with uncorrected Fisher’s LSD. *P < 0.05. **c**, Growth curves of tumours from male mice treated with vehicle, RANO alone 50 mg/kg, daily, BRAFi alone (25 mg/kg, daily) or with BRAFi (25 mg/kg, daily) and RANO (50 mg/kg, daily), RANO was added at day 23 when BRAFi treated tumours showed a reduced response to treatment. Data are presented as mean ± s.e.m. based on *n* = 13 tumours in vehicle group, 14 tumours in RANO and BRAFi/RANO group and 16 in the BRAFi group. **d**,**e**, Growth curves of individual tumours from male mice treated with (**d**) BRAFi alone or (**e**) with BRAFi and RANO as described in (**c**). **f**, Kaplan-Meier plots of progression free survival of male mice treated as indicated. Progression was declared when tumours exceeded a volume of twice the volume at day 8, when BRAFi was added (dashed line). Log-rank (mantel Cox) test: BRAFi vs BRAFi/RANO HR = 2.58. **g**, Tumour end volume of mice treated as indicated in (**c**) at days 34 and 35 of treatment. Data are from *n* = 14 tumours and presented as mean ± s.e.m. analysed by one-way ANOVA with uncorrected Fisher’s LSD. **P < 0.01. **h** qPCR analysis of the indicated genes in A375 tumours from mice treated as indicated. Data are triplicates from *n* = 8 tumours for vehicle or BRAFi, *n* = 4 tumours for RANO responder (resp) or RANO progressed (prog), respectively. Data and are presented as mean ± s.e.m. analysed by one-way ANOVA with uncorrected Fisher’s LSD. *P < 0.05; **P < 0.01; ***P < 0.001. Source data

Because the findings from our in vitro system appeared to reliably reflect the situation in patients, we next tested the efficacy of RANO in vivo. Previous reports have shown that 3 weeks of ETO treatment increases the growth of A375 tumours in mice^11^. However, when we treated mice with RANO for 4 weeks, A375 tumour growth was unaltered (Extended Data Fig. 4a). To further assess whether RANO could improve the response to BRAFi in vivo, we treated mice with vemurafenib until the first tumours stopped responding. At this point, mice were randomized and while vemurafenib treatment was kept, one group received a supplement of vehicle, and the other group was treated with RANO. Strikingly, the addition of RANO significantly reduced tumour growth, delayed the onset of resistance to vemurafenib and increased progression-free survival (Fig. 3c,d and Extended Data Fig. 4a,b). These observations were independent of the sex of the mice, as we obtained comparable data from females (Fig. 3c,d and Extended Data Fig. 4a,b) and males (Extended Data Fig. 4c–g).

As observed in patients as well as in A375VR cells, tumours that had progressed on BRAFi expressed increased levels of FAO regulators (Fig. 3e). In tumours that were still growth inhibited in the presence of RANO, the increase in FAO regulator expression was generally reverted, but intriguingly this was not observed in tumours that had progressed on combined BRAFi/RANO treatment (Fig. 3e). Some glycolysis regulators displayed slightly reduced expression in BRAFi-progressed tumours, and while there was no clear trend in RANO responders, their expression was restored in BRAFi/RANO-progressed tumours (Fig. 3e). BRAFi-progressed tumours appeared to have reactivated MAPK signalling as *DUSP6* expression was not suppressed (Fig. 3f). Effective suppression was however seen in BRAFi/RANO responders, but in BRAFi/RANO-progressed tumours *DUSP6* expression had fully recovered (Fig. 3f). Overall, this suggests that tumours that have reinstated MAPK signalling can overcome BRAFi treatment but to evade the BRAFi/RANO combination treatment tumours reinstate glycolysis and upregulate FAO regulators.

BRAFi-progressed tumours displayed heterogeneity with regard to melanoma state markers with distinct populations expressing high or low levels of the respective markers (Extended Data Fig. 4h). Remarkably, while in RANO-responding tumours these changes were moderated, a slight re-establishment of separated melanoma state markers could be observed in BRAFi/RANO-progressed tumours (Extended Data Fig. 4h). In line with all the observed changes, melanoma cells isolated from progressed BRAFi or BRAFi/RANO tumours showed resistance to BRAFi in vitro (Fig. 3g), and moreover, cells from BRAFi/RANO-progressed tumours were also resistant to RANO (Fig. 3g).

### Ranolazine diminishes the neural crest stem cell state in BRAFi-resistant melanoma

**Fig. 4:**
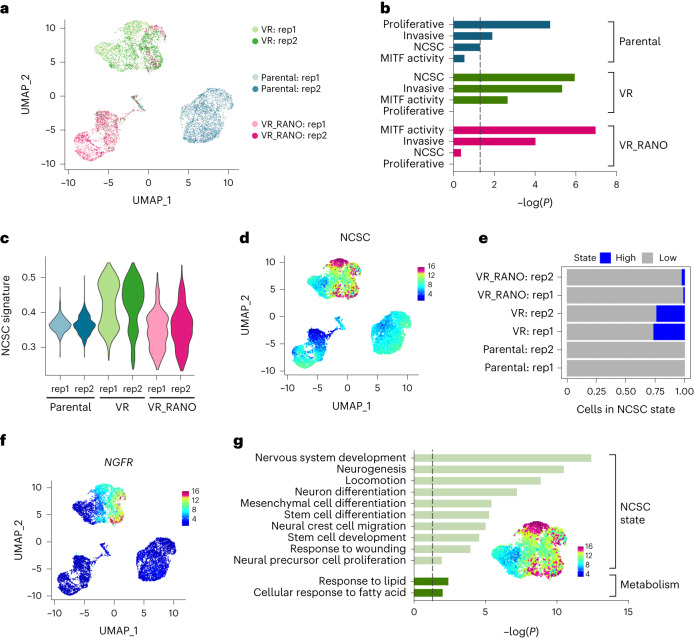
Ranolazine diminishes the neural crest stem cell state. **a**, Uniform manifold approximation and projection (UMAP) visualization of 10,133 cells coloured by treatment groups and replicate. **b**, Melanoma cell states according to Rambow et al.^6^ enriched (hypergeometric tests) in the top gene markers of each treatment group. The dashed line indicates *P* = 0.05. **c**, NCSC signature scores represented by violin plots. **d**, UMAP plot coloured by the expression of the NCSC state. **e**, Discrete expression of the NCSC state based on the 90th percentile of signature scores across the whole dataset. **f**, UMAP plot coloured by the expression of *NGFR*. **g**, GOBP terms enriched (hypergeometric tests) in the subset of genes (top 200) most highly correlated to the NCSC signature score. A UMAP plot of the VR compartment coloured for the NCSC signature is indicated. The dashed line indicates *P* = 0.05.

**Extended Data Fig. 5:**
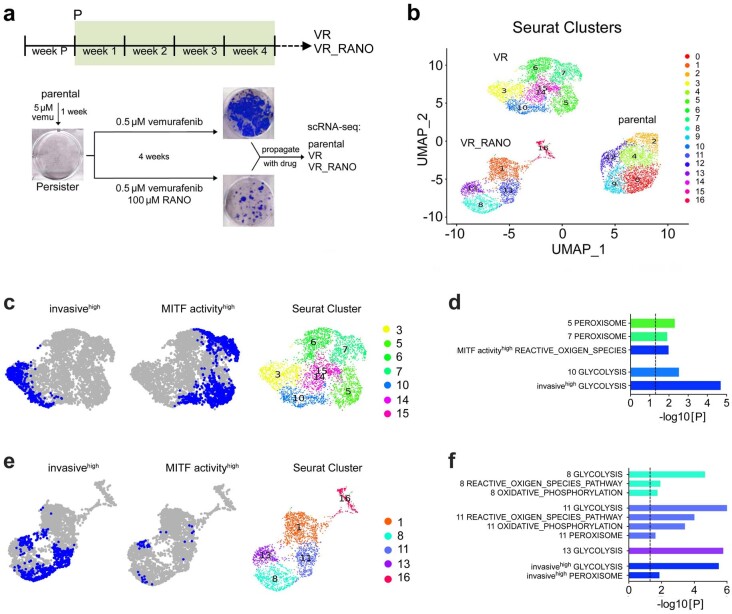
Ranolazine alters the transcription state of BRAFi resistant melanoma cells. **a**. Protocol for the generation of single-cell RNAseq data. **b**, UMAP of Seurat clusters identified in the individual treatment groups parental, VR and VR_RANO. **c**, UMAP plots of VR compartment coloured for high discrete expression of the invasive or MITF activity state based on the 90th percentile of signature scores across the whole dataset. The corresponding clusters are shown. **d**, Enrichment analysis using gene markers for invasive^high^ (VR cells), MITF activity^high^ (VR cells) and indicated clusters (VR compartment) against MSigDB hallmark gene set collection^63^. **e**, UMAP plots of VR_RANO compartment coloured for high discrete expression of the invasive or MITF activity state based on the 90th percentile of signature scores across whole dataset. The corresponding clusters in VR_RANO compartment are shown. **f**, Enrichment analysis using gene markers for invasive^high^ (VR_RANO cells), MITF activity^high^ (VR_RANO cells) and indicated clusters (VR_RANO compartment) against MSigDB hallmark gene set collection^63^.

To better understand how RANO modulates the acquisition of BRAFi resistance at the molecular level, we performed single-cell RNA-sequencing (scRNA-seq) in parental A375 cells, and cells selected for resistance to vemurafenib alone (VR) or vemurafenib in the presence of RANO (VR_RANO; Extended Data Fig. 5a). Two-dimensional representation of the individual melanoma cell transcriptomes in two independent experiments identified three disconnected compartments corresponding almost entirely to parental, VR or VR_RANO cells (Fig. 4a) and each population was characterized by specific clusters (Extended Data Fig. 5b).

Single-cell analysis allowed us to assess whether RANO impacts on individual transcriptional melanoma states by modulating metabolic activities, and as such could influence the outcome of acquired resistance. To characterize the main three populations, we performed enrichment analysis (hypergeometric test) comparing their top gene markers with those of the transcriptional states previously described by Rambow et al.^6^ and found that the ‘proliferative’, ‘invasive’, ‘MITF activity’ and ‘NCSC’ states were significantly enriched in individual cell populations (Fig. 4b). In parental cells, the proliferative state dominated, but in BRAFi-acquired resistant VR cells, invasive, NCSC and MITF activity states were significantly enriched (Fig. 4b). Intriguingly, the NCSC state, detectable in VR cells, was not significantly enriched in VR_RANO cells (Fig. 4b–e). This was mirrored in a RANO-induced depletion of cells expressing the NCSC marker NGFR (Fig. 4f), a membrane receptor that confers resistance not only to MAPKi but also to immunotherapy^9^.

To understand the mechanisms underlying the NCSC state in VR cells, we identified a core gene expression programme with the most highly correlated genes to the NCSC signature and performed enrichment analysis against known biological functions (Fig. 4g). This set overlapped with genes associated to NCSC-related traits and fatty acid metabolism (Fig. 4g). No significant enrichment for any other pathway linked to energy metabolism was detected, which might explain why cells of this melanoma state are particularly sensitive to RANO.

### Ranolazine rewires metabolism in BRAFi-resistant melanoma

**Fig. 5:**
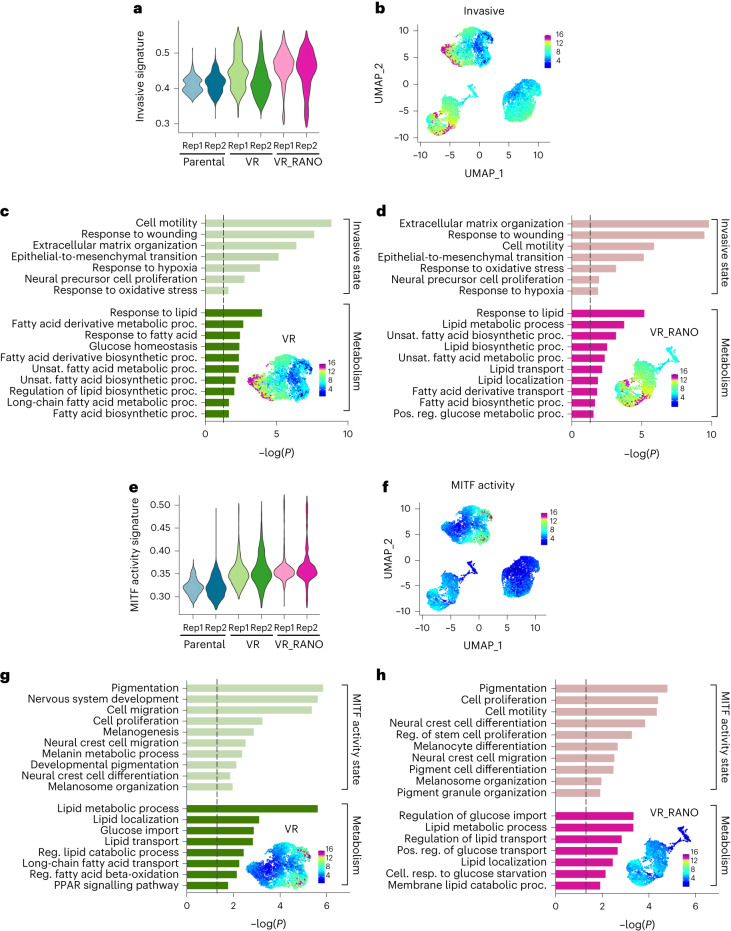
Ranolazine alters the metabolic state of BRAFi-resistant melanoma cells. **a**, Invasive state signature scores represented by violin plots. **b**, UMAP plot coloured by the expression of the invasive state. **c,d**, GOBP terms enriched (hypergeometric tests) in the subset of genes (top 200) most highly correlated to the invasive signature scores in VR (**c**) or VR_RANO (**d**) cells. The dashed line indicates *P* = 0.05. Relevant UMAP plots coloured for the respective melanoma states are indicated. **e**, MITF activity state signature scores represented by violin plots. **f**, UMAP plot coloured by the expression of the MITF activity state. **g**,**h**, GOBP terms enriched (hypergeometric tests) in the subset of genes (top 200) most highly correlated to the MITF activity signature scores in VR (**g**) or VR_RANO (**h**) cells. The dashed line indicates *P* = 0.05. Relevant UMAP plots coloured for the respective melanoma states are indicated.

The invasive state signature was detectable in the parental compartment but was enriched in the VR and the VR_RANO compartment (Fig. 5a,b). As expected, the invasive signature core gene expression programme was characterized by GO terms linked to migration and neural precursor cell proliferation but was also enriched in functions related to fatty acid biosynthesis, and this was seen in both the VR and the VR_RANO compartments (Fig. 5c,d).

As with the invasive state, the MITF activity state was enriched in the VR as well as VR_RANO compartments, when compared to parental cells (Fig. 5e,f). Fatty acid metabolism also dominated the MITF activity state in the VR compartment, but it was linked to lipid catabolism, FAO and PPAR signalling (Fig. 5g), which is entirely in line with high MITF activity controlling oxidative phosphorylation through PPARGC1A in BRAF-mutant melanoma^13^. In contrast, in the VR_RANO compartment, MITF activity markers were less enriched in fatty acid metabolism, but functions related to glucose import and transport were presented with strong statistical evidence (Fig. 5h).

In the VR compartment, the invasive state was expressed in a group of cells almost mutually exclusive to the MITF activity state (Fig. 5b,c,f,g). This was particularly noticeable when selecting subpopulations with the most highly expressed cells, defined as invasive^hi^ and MITF activity^hi^, respectively (Extended Data Fig. 5c). MITF activity^hi^ state cells were contained within Seurat clusters 5 and 7 (Extended Data Fig. 5b,c), and were enriched for the Hallmarks peroxisome and reactive oxygen species (ROS) (Extended Data Fig. 5d). On the other hand, cluster 10 and invasive^hi^ markers were enriched for glycolysis (Extended Data Fig. 5d). Overall, this suggests that in VR cells two distinct populations of cells coexist, and while fatty acid metabolism appears central to their metabolic state, one population (invasive state) drives fatty acid anabolism whereas the other (MITF activity state) uses their catabolism.

In the VR_RANO compartment, the MITF activity signature score was expressed more universally at medium level, when compared to the VR compartment (Fig. 5e,f). In fact, while invasive^hi^ state cells were readily seen in the VR_RANO compartment, MITF activity^hi^ cells were hardly detectable (Extended Data Fig. 5e). Enrichment analysis showed glycolysis, oxidative phosphorylation and ROS as some of the most represented hallmarks of invasive^hi^ co-located VR-RANO clusters (8, 11 and 13; Extended Data Fig. 5f). Thus, overall, RANO establishes populations of cells, which appear to drive fatty acid anabolism and use glycolysis and oxidative phosphorylation to meet energy demands.

### Ranolazine affects interferon and methionine metabolism-related transcriptomics

**Fig. 6:**
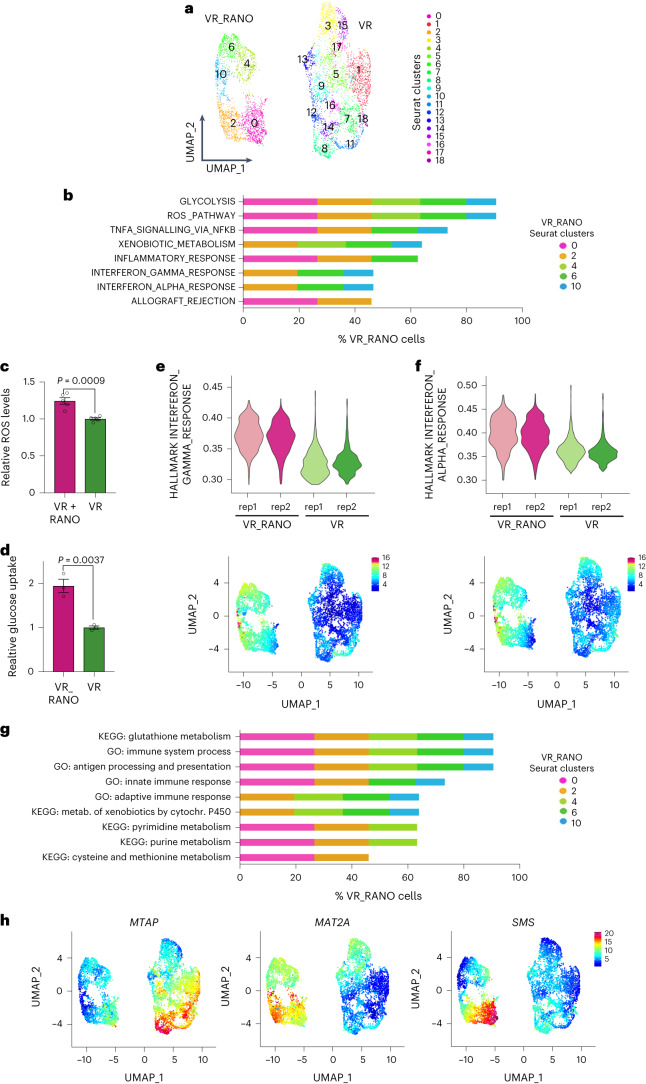
Interferon signalling and methionine metabolism are enriched in ranolazine-treated BRAFi-resistant melanoma cells. **a**, UMAP visualization of 6,457 VR and VR_RANO cells coloured by Seurat clusters. **b**, Hypergeometric test for enrichment analysis using cluster gene markers against the MSigDB hallmark gene-set collection. Only VR_RANO clusters enriched at significance level of *P*_adj_ < 0.05 are indicated. The percentage of VR_RANO cells distributed in the respective VR_RANO main clusters is indicated for each hallmark. **c**,**d** Quantification of cellular ROS levels in VR cells in the presence or absence of RANO and of glucose uptake in VR and VR_RANO cells. Data are presented as the mean ± s.e.m. based on *n* = 5 (**c**) and *n* = 3 (**d**) biological replicates analysed by two-tailed unpaired *t*-test. 1. **e**,**f**, Hallmarks (**e**) interferon gamma response and interferon alpha response (**f**) signature scores in VR_RANO or VR cells represented by violin plot (top) and UMAP (bottom). **g**, Hypergeometric test for enrichment analysis using cluster gene markers against the KEGG pathway and GOBP gene-set collections. Only VR_RANO clusters enriched at significance level of *P* < 0.05 are indicated. The percentage of VR_RANO cells distributed in the respective VR_RANO main clusters is indicated for each term. **h**, UMAP visualization coloured by *MTAP*, *MAT2A* and *SMS* expression. Source data

**Extended Data Fig. 6:**
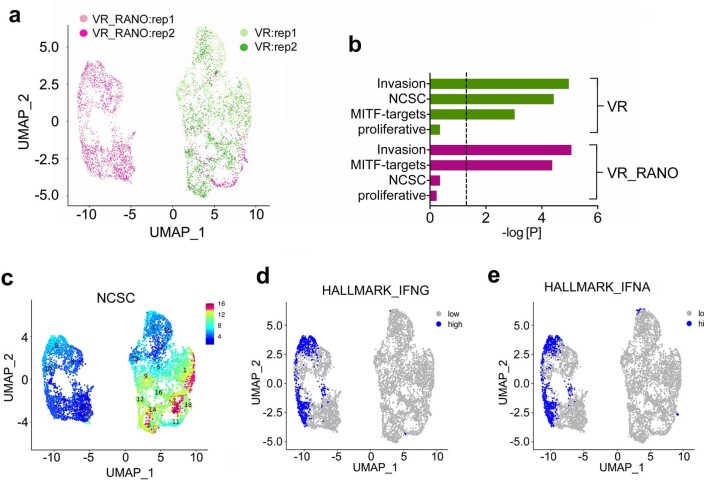
Ranolazine enhances interferon signalling in BRAFi resistant melanoma cells. **a**, Uniform Manifold Approximation and Projection (UMAP) visualisation of 6,457 cells coloured by treatment and replicate groups. **b**, Melanoma cell states according to Rambow et al^6^ enriched (hypergeometric tests) in treatment’s gene markers. The dashed line indicates P = 0.05. **c**, UMAP visualisation of VR and VR_RANO cells coloured by the NCSC state^6^ signature score **d,e**, UMAP visualisation of VR and VR_RANO cells coloured for high versus low discrete states (based on the 90th percentile) of the Hallmark signatures (**d**) interferon gamma signalling and (**e**) interferon alpha signalling.

To reveal more detail of the effect of RANO on the response of melanoma cells to BRAFi, we focused on the VR and VR_RANO populations. Two-dimensional representation showed two disconnected components corresponding to each condition (Extended Fig. 6a). We could confirm that the NCSC state, otherwise observed in VR cells, was not significantly enriched in VR_RANO cells (Extended Data Fig. 6b,c). Each population was characterized by specific clusters (Fig. 6a). Enrichment analysis of the individual clusters identified the Hallmark terms glycolysis and ROS to be enriched in all clusters of VR_RANO cells (Fig. 6b). An increase in cellular ROS levels and glucose uptake could be confirmed at the functional level in VR_RANO cells (Fig. 6c,d).

Remarkably, in the VR_RANO clusters, many of the additional enriched hallmarks were associated with immunity, such as TNFA, IFNA and IFNG signalling as well as inflammatory response and allograft rejection (Fig. 6b). Accordingly, genes linked to IFNA or IFNG response were expressed at higher levels in RANO-treated cells as compared to VR cells (Fig. 6e,f and Extended Data Fig. 6d,e). Changes in immunity were also detected using the Gene Ontology Biological Processes (GOBP) gene-set collection with genes associated with antigen presentation, innate and adaptive immune response enriched in VR_RANO clusters (Fig. 6g). The KEGG pathway gene-set collection revealed that RANO treatment induced enrichment of terms related to glutathione, pyrimidine and purine metabolism (Fig. 6g), all three of which are linked to the methionine cycle. The latter is part of the ‘cysteine and methionine metabolism’, which was also significantly enriched (Fig. 6g). In line with this, we found that key enzymes that act either in the methionine cycle (MAT2A) or in the methionine salvage pathway (SMS, MTAP) depicted subpopulations of cells with profound differential presence in the VR and VR_RANO conditions (Fig. 6h). In particular, the expression of *MTAP* and *MAT2A* appeared mutually exclusive, with *MTAP* subpopulations profoundly reduced and *MAT2A* subpopulations enriched in the RANO cluster component (Fig. 6h).

### Ranolazine modulates methionine metabolism

**Fig. 7:**
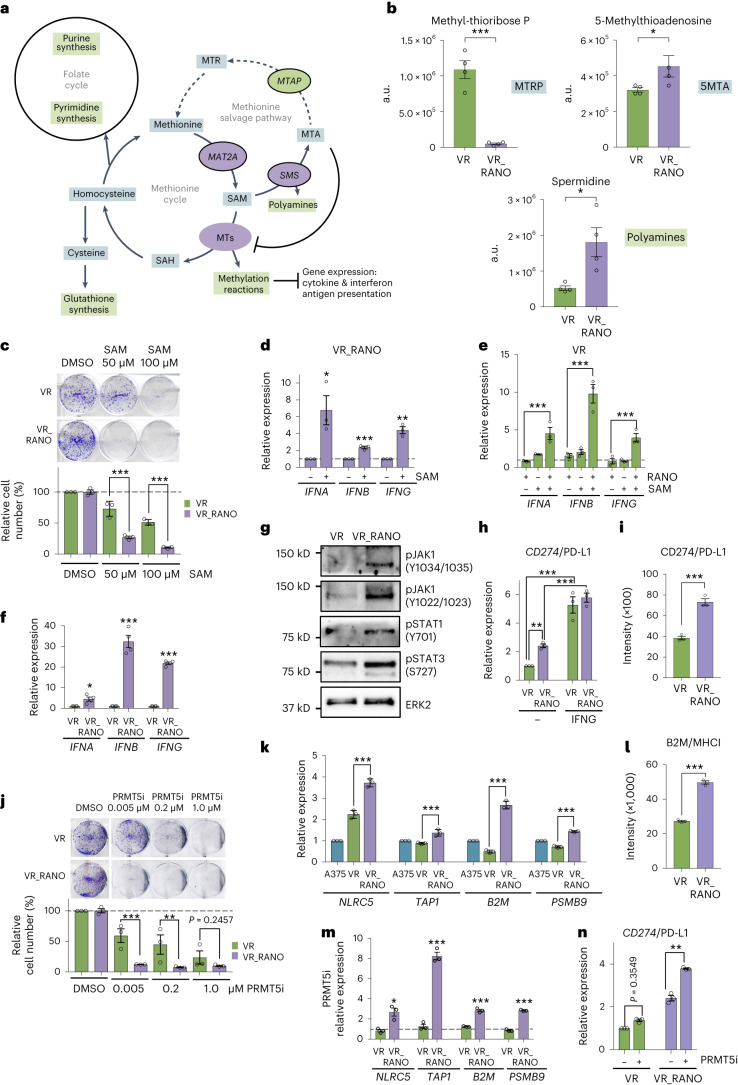
Altered methionine metabolism suppresses PRMT5 activity in VR_RANO cells. **a**, Scheme showing methionine metabolism linked to the folate cycle and glutathione synthesis. The genes enriched (*MAT2A*, *SMS*) and suppressed (*MTAP*) in VR_RANO cells are indicated. **b**, Metabolomics analyses showing reduced levels (integrated peak areas, a.u.) of MTRP and increases in 5MTA and spermidine in VR_RANO cells compared to VR. Data are presented as the mean ± s.e.m. based on *n* = 4 biological replicates and analysed by two-tailed unpaired *t*-test. *P < 0.05; ***P < 0.001. **c**, CFA quantification of VR or VR_RANO cells treated with 50 µM or 100 µM SAM. Data are presented as the mean ± s.e.m. based on *n* = 3 biological replicates analysed by one-way ANOVA with uncorrected Fisher’s LSD. **P < 0.01; ***P < 0.001. **d**, qPCR analysis of the indicated genes in VR_RANO cells treated with 50 µM SAM for 8 h. Data are presented as the mean ± s.e.m. based on *n* = 3 biological replicates and analysed by two-tailed unpaired *t*-test. *P < 0.05; **P < 0.01; ***P < 0.001. The dashed line at 1.0 indicates untreated cells. **e**, qPCR analysis of the indicated genes in VR cells treated with 50 µM SAM for 8 h alone or in combination with RANO. Data are presented as the mean ± s.e.m. based on *n* = 3 biological replicates analysed by one-way ANOVA with uncorrected Fisher’s LSD. ***P < 0.001. The dashed line at 1.0 indicates untreated cells. **f**, qPCR analysis of the indicated genes in VR and VR_RANO cells. Data are presented as the mean ± s.e.m. based on *n* = 4 biological replicates by two-tailed unpaired *t*-test. *P < 0.05; ***P < 0.001. **g**, Western blot for the indicated proteins in VR and VR_RANO cells representative for *n* = 3 repeated experiments with similar results. ERK2 served as the loading control. **h**, qPCR analysis of *CD274*/PD-L1 in VR and VR_RANO cells in the absence or presence of IFNG. Data are presented as the mean ± s.e.m. based on *n* = 3 biological replicates analysed by one-way ANOVA with uncorrected Fisher’s LSD. **P < 0.01. **i**, Flow cytometry analysis of CD274 expression on VR or VR_RANO cells. Data are presented as the mean ± s.e.m. based on *n* = 3 biological replicates and analysed by two-tailed unpaired *t*-test. ***P < 0.001. **j**, Quantification of VR or VR_RANO cells treated with the indicated concentrations of the PRMT5 inhibitor GSK3326595 (PRMT5i). Data are presented as the mean ± s.e.m. based on *n* = 3 biological replicates analysed by one-way ANOVA with uncorrected Fisher’s LSD. ***P < 0.001. **k**, qPCR analysis of the indicated genes in A375, VR and VR_RANO cells. Data are presented as the mean ± s.d. based on *n* = 3 technical replicates analysed by one-way ANOVA with uncorrected Fisher’s LSD. **P < 0.01; ***P < 0.001. **l**, Flow cytometry analysis of B2M expression on VR or VR_RANO cells. Data are presented as the mean ± s.e.m. based on *n* = 3 biological replicates and analysed by two-tailed unpaired *t*-test. ***P < 0.001. **m**, qPCR analysis of the indicated genes in VR and VR_RANO cells treated with GSK3326595. Data are presented as the mean ± s.e.m. based on *n* = 3 biological replicates and analysed by two-tailed unpaired *t*-test. *P < 0.05; ***P < 0.001. The dashed line at 1.0 indicates untreated cells. **n**, qPCR analysis of *CD274*/PD-L1 in VR and VR_RANO cells in the absence or presence of GSK3326595. Data are presented as the mean ± s.e.m. based on *n* = 3 biological replicates analysed by one-way ANOVA with uncorrected Fisher’s LSD. **P < 0.01. Source data

Methionine metabolism is central to cellular processes such as methylation reactions, glutathione synthesis and the folate cycle^29^. Apart from its uptake or its synthesis from homocysteine, cells can recycle methionine in the methionine salvage pathway. Therefore, MTAP synthesizes methionine from methyl-thioadenosine (MTA), which is a by-product of the *S*-adenosylmethionine (SAM)-dependent polyamine biosynthesis in which SMS takes part; SAM itself is produced by MAT2A from methionine (Fig. 7a).

Our scRNA-seq analysis had identified increased expression of *MAT2A* and *SMS* and reduced *MTAP* expression in VR_RANO cells (Fig. 6h). Consistent with such changes, we observed reduced amounts of methyl-thioribose phosphate (MTRP) and increased amounts of MTA and spermidine in VR_RANO compared to VR cells (Fig. 7b). These metabolic alterations suggest that the increased expression of *SMS* in VR_RANO cells produces more MTA from SAM, and MTA then accumulates due to the reduced expression of MTAP. To further test this idea, we added exogenous SAM, which should further increase MTA accumulation in MTAP^lo^ VR_RANO cells. Importantly, accumulated MTA can act as a natural competitor of SAM and inhibit methyltransferases (Fig. 7a), leading to altered epigenetics and reduced cell growth^29–31^. In line with our hypothesis MTAP^lo^ VR_RANO cells were significantly more responsive to SAM addition than VR cells when cell growth was assessed (Fig. 7c).

Considering the correlation of methionine metabolism regulators and interferon signalling in VR_RANO cells (Fig. 6), we tested whether SAM, through MTA accumulation, could alter epigenetics and impact on the expression of interferons. Indeed, SAM alone was able to significantly upregulate *IFNA*, *IFNB* and *IFNG* expression in MTAP^lo^ VR_RANO cells (Fig. 7d). In VR cells, treatment with SAM for 8 h alone only caused a weak induction of interferon expression, which was however significantly amplified by the addition of RANO (Fig. 7e). Furthermore, in VR_RANO cells, in which MTA levels are higher (Fig. 7b), the basal expression of *IFNA*, *IFNB* and *IFNG* was higher compared to VR cells (Fig. 7f), which correlated with elevated interferon signalling, detectable by an increase in phosphorylated JAK1, STAT1 and STAT3 (Fig. 7g). Finally, the immune checkpoint regulator *CD274* (PD-L1), whose expression can be induced by interferon signalling^32^, was also increased in MTAP^lo^ VR_RANO cells (Fig. 7h,i).

In cancer cells, the *MTAP* gene is frequently co-deleted with *CDKN2A*, and this results in MTA-mediated inhibition of the methyltransferase PRMT5; in line with this, the sensitivity to PRMT5 inhibitors is higher in cells with increased MTA levels^33–35^. As seen with SAM, VR_RANO cells were significantly more sensitive to the PRMT5 inhibitor GSK3326595 (Fig. 7j), suggesting that RANO, which increases MTA levels, impacts on PRMT5 activity. PRMT5 suppresses the expression of NLCR5 (ref. ^36^), which is the master regulator of major histocompatibility complex (MHC) class I-related genes involved in antigen presentation^37,38^. Because VR_RANO cells were enriched for genes linked to antigen presentation (Fig. 6g), we assessed the expression of *NLRC5* and its targets and found increased expression in VR_RANO cells (Fig. 7k,l); this expression could be further enhanced by GSK3326595 (Fig. 7m), corroborating the involvement of PRMT5. PRMT5 has also been shown to directly regulate the expression of PD-L1 (ref. ^39^) and we could confirm that GSK3326595 specifically increased *CD274* (PD-L1) expression in VR_RANO cells (Fig. 7n).

### Ranolazine improves anti-PD-L1 immunotherapy

**Fig. 8:**
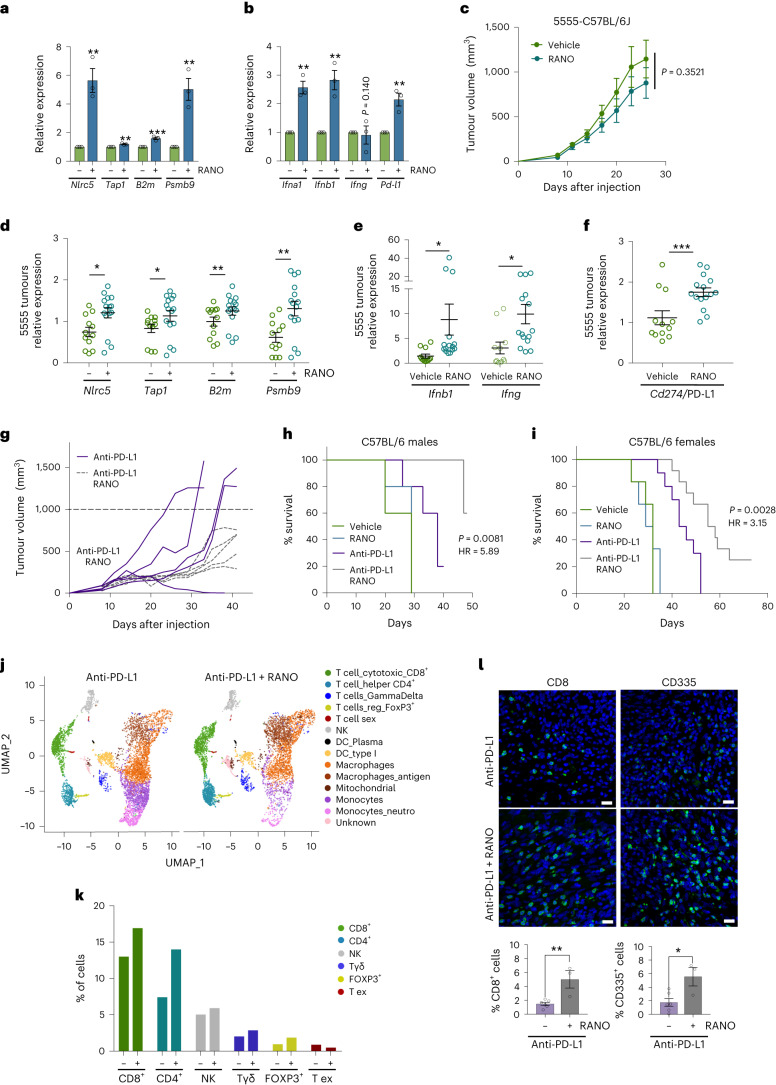
Ranolazine induces an immunogenic signature and improves anti-PD-L1 therapy in mice. **a,b**, qPCR analysis of the indicated genes in BRAF^V600E^ 5555 mouse melanoma cells. Data are presented as the mean ± s.e.m. based on *n* = 3 biological replicates and analysed by two-tailed unpaired *t*-test. **P < 0.01; ***P < 0.001. **c**, Tumour growth curves of male C57BL/6 mice treated with vehicle or RANO (300 mg per kg body weight). *n* = 5 mice in each group. **d**–**f**, qPCR analysis of the indicated genes in BRAF^V600E^ 5555 melanomas from mice treated with vehicle or RANO (300 mg per kg body weight daily) for 29 d. Data are presented as the mean ± s.e.m. based on *n* = 3 technical replicates of tumours from four vehicle-treated mice and from five RANO-treated mice and were analysed by two-tailed unpaired *t*-test. *P < 0.05; **P < 0.01; ***P < 0.001. **g**, Growth curves of individual tumours from male C57BL/6 mice treated with anti-PD-L1 (10 mg per kg body weight, every 3 d) or anti-PD-L1 and RANO (300 mg per kg body weight, daily) starting at day 6. *n* = 5 tumours in each group. **h**, Kaplan–Meier plots of survival of male mice treated as indicated. Mice with a tumour that exceeded a volume of 1,000 mm^3^ (dashed line in **g**) were declared to have reached the endpoint of the experiment (death). Log-rank (Mantel–Cox) test was performed for anti-PD-L1/anti-PD-L1 RANO. **i**, Kaplan–Meier plots of survival of female C57BL/6 mice treated as indicated in Extended Data Fig. 6e–h. Mice with a tumour that exceeded a volume of 1,000 mm^3^ were declared to have reached the endpoint of the experiment (death). Log-rank (Mantel–Cox) test was performed for anti-PD-L1/anti-PDL1 RANO. **j**, UMAP representing CD45^+^ cells isolated from BRAF^V600E^ 5555 melanomas from male mice after 17 d of treatment. Individual cell populations were annotated using lineage-specific signatures. **k**, Percentage of annotated lymphocytic populations within the tumour immune infiltrate comparing tumours from mice treated with anti-PD-L1 alone or with anti-PD-L1 and RANO. **l**, Immunofluorescence analysis for CD8 and CD335 of tumours 16 d after treatment with anti-PD-L1 in the absence or presence of RANO. Data are presented as the mean ± s.e.m. based on *n* = 6 or *n* = 3 biological replicates, respectively and analysed by two-tailed unpaired *t*-test. *P < 0.05; **P < 0.01. Source data

**Extended Data Fig. 7:**
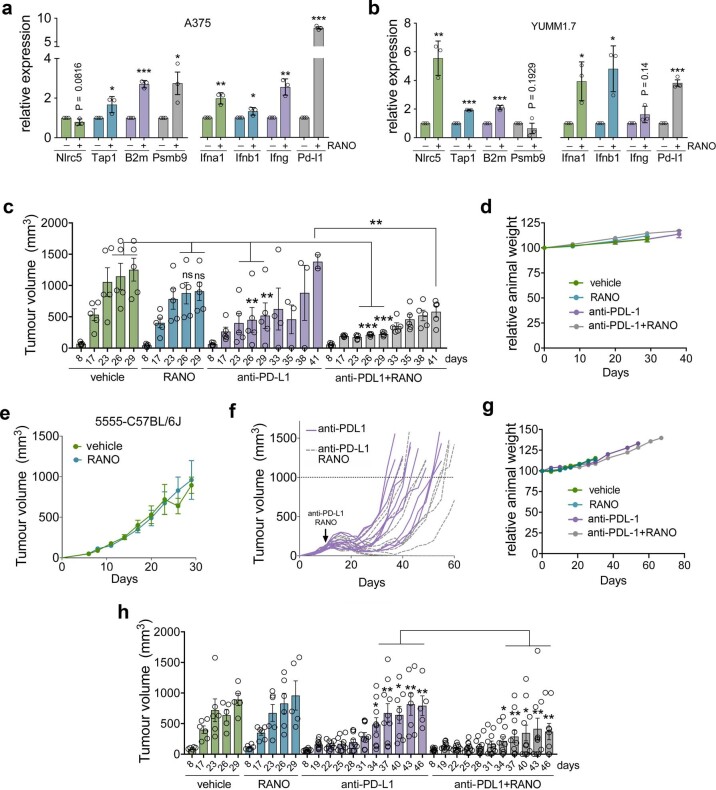
Ranolazine improves anti-PD-L1 therapy. **a,b**, mRNA expression of the indicated genes in (**a**,) A375 human melanoma cells and in (**b**) YUMM1.7 mouse melanoma cells after treatment with RANO. Expression in untreated cells was set 1. Data are presented as mean ± s.e.m. based on *n* = 3 biological replicates and analysed by two-tailed unpaired *t*-test. *P < 0.05; **P < 0.01; ***P < 0.001. **c**, Tumour volume at the indicated days in male C57BL/6 mice treated with vehicle, RANO (300 mg/kg, daily), anti-PD-L1 (10 mg/kg, every 3 days) or anti-PD-L1 and RANO (300 mg/kg, daily) starting at day 6. Data are presented as mean ± s.e.m. based on *n* = 5 mice in each group and analysed by one-way ANOVA with uncorrected Fisher’s LSD. **P < 0.01; ***P < 0.001; *P* = 0.2245; *P* = 0.1194. **d**, Relative animal weight of mice related to (**c**). Data are presented as mean ± s.e.m. **e**, Tumour growth curves of female C57BL/6 mice treated with vehicle or RANO (300 mg/kg/daily). Data are presented as mean ± s.e.m. based on *n* = 5 mice in each group. **f**, Growth curves of individual tumours from female C57BL/6 mice treated with anti-PD-L1 (10 mg/kg, every 3 days) or anti-PD-L1 and RANO (300 mg/kg, daily) starting at day 6. *n* = 10 tumours for anti-PD-L1 and 11 for the combination treatment. **g**, Relative animal weight of mice related to (**e,f**). Data are presented as mean ± s.e.m. **h**, Tumour volume at the indicated days in female C57BL/6 mice treated as described in (**e,f**). Data are presented as mean ± s.e.m. based on 10 tumours for anti-PD-L1 and 11 for the combination treatment, analysed by one-way ANOVA with uncorrected Fisher’s LSD. **P < 0.01; ***P < 0.001. Source data

Our results suggest that the FDA-approved drug RANO can impact on tumour immunity by inducing the upregulation of antigen presentation, interferon signalling and PD-L1 expression. Importantly, we could confirm that RANO can induce this immunogenic phenotype as a single agent in the absence of vemurafenib, because it induced the expression of antigen presentation genes, interferons and PD-L1 in A375 cells and in BRAF^V600E^ mouse melanoma cell lines 5555 and YUMM1.7 (Fig. 8a,b and Extended Data Fig. 7a,b). When C57BL/6J mice bearing 5555 melanomas were treated with RANO, this resulted in a slight but not significant reduction in tumour volume without any impact on animal weight (Fig. 7c and Extended Data Fig. 6c,d). These RANO-treated tumours displayed increased expression of antigen presentation genes, as well as *Ifnb1*, *Ifng* and *Cd274* (Fig. 8d–f), indicating that RANO is effective in inducing an immunogenic phenotype in vivo.

Given these immunogenic changes induced by RANO, we assessed whether it would sensitize melanoma cells in vivo to PD-L1 targeting therapy. The 5555 melanoma-bearing C57BL/6J mice were treated with anti-PD-L1 in the absence or presence of RANO. While anti-PD-L1 monotherapy produced a varied response throughout treatment, addition of RANO potentiated the response (Fig. 8g). Moreover, while anti-PD-L1-treated tumours progressed within 30 d after the initiation of treatment, tumour growth in RANO/anti-PD-L1-treated mice was significantly reduced (Fig. 8g and Extended Data Fig. 7c). Most importantly, the survival of mice treated with RANO/anti-PD-L1 was significantly better than that of mice treated only with anti-PD-L1 monotherapy (Fig. 8h). These observations were independent from the sex of the mice, as we obtained comparable data from males (Fig. 8c,g,h and Extended Data Fig. 7c,d) and females (Fig. 8i and Extended Data Fig. 7e–h).

**Extended Data Fig. 8:**
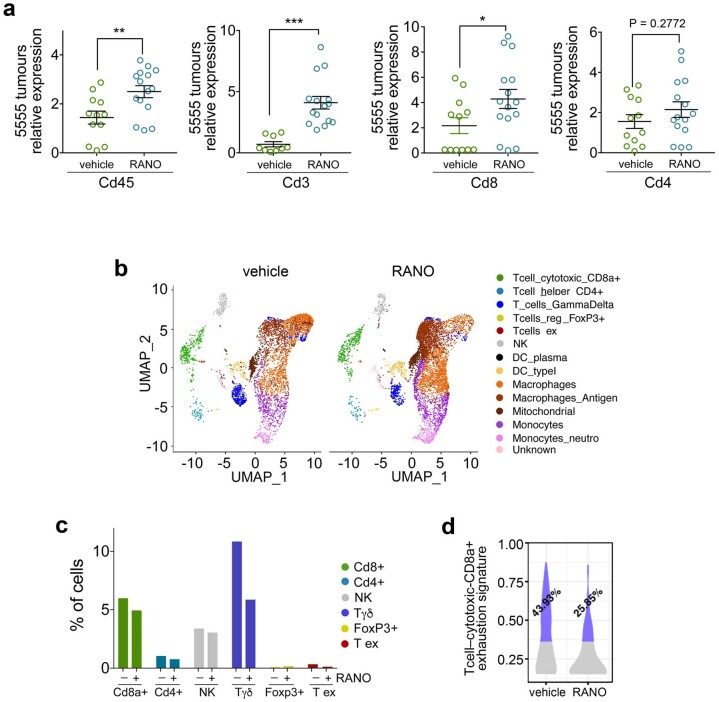
Ranolazine increases abundance and reduces exhaustion of Cd8+ T cells. **a**, qPCR analysis of the indicated genes in 5555 mouse melanoma tumours from mice treated for 21 days. Data and are presented as mean ± s.e.m. analysed by by two-tailed unpaired *t*-test. *P < 0.05; **P < 0.01; ***P < 0.001. **b**, UMAP representing CD45+ cells isolated from 5555 melanomas from male mice after 13 days of treatment. Individual lymphocytic cell populations were annotated using lineage-specific signatures. **c**, Percentage of annotated lymphocytic populations within the tumour immune infiltrate comparing tumours from mice treated with vehicle or with RANO. **d**, Violin plots representing the enrichment of the exhaustion signature in CD8 ^+^ -T cells isolated from mice treated for 13 days with vehicle or RANO. Source data

To assess the effect of RANO on the immune microenvironment in the context of anti-PD-L1 therapy, we treated mice with anti-PD-L1 alone or in combination with RANO for 17 d. scRNA-seq of immune cell isolates from these tumours identified a notable rise in CD8^+^ cytotoxic T cells and CD4^+^ helper T cells, as well as a slight increase in natural killer (NK) cells (Fig. 8j,k). Immunostaining of tumours collected after 16 d of treatment corroborated the increase in CD8^+^ T cell and NK cell abundance (Fig. 8l). This suggested that RANO enhances immune cell infiltration by increasing the immunogenicity of melanoma cells. In line with this, we observed that tumours isolated from mice treated with RANO alone for 21 d expressed higher levels of *Cd45*, *Cd3g* and *Cd8*; a similar trend was observed with *Cd4*, which did however not reach significance (Extended Data Fig. 8a). When we assessed an earlier time point of treatment with RANO alone (13 d) through scRNA-seq analysis of the immune compartment, we observed no increase in CD8^+^ or CD4^+^ T cell or NK cell abundance, and rather a slight reduction (Extended Data Fig. 8b,c). Intriguingly, however, treatment with RANO reduced the expression of genes associated with T cell exhaustion in CD8^+^ cells (Extended Data Fig. 8d and Supplementary Table 1), which could contribute to increased CD8^+^ T cell abundance at a later stage. Thus, our data suggest that increased immune cell infiltration as well as reduced CD8^+^ T cell exhaustion is part of the RANO activity that improves the efficacy of anti-PD-L1 treatment.

## Discussion

In this study, we have focused on the interplay between fatty acid catabolism and melanoma cell response to first-line treatments. We show that beyond the initial response to BRAFi, which involves intense metabolic rewiring^10–12,15^, FAO is relevant for survival once melanoma cells have acquired full BRAFi resistance. Inhibiting FAO with RANO delayed the appearance of acquired resistant tumours and improved progression-free survival in mice. Upregulation of FAO regulator genes was seen in acquired resistant melanoma cells in vitro and in vivo in tumour xenografts, but most importantly in tumours from patients progressed on MAPKi. At this stage, despite still displaying a certain degree of intra-tumour heterogeneity, tumours have frequently settled for a distinct melanoma transcriptional state, which can impact on further treatment options^5,40,41^. Intriguingly, we found that the increase in FAO regulators frequently observed in relapsed tumours from melanoma patients correlated with multiple melanoma state markers, implying a general relevance of lipid metabolism for acquired resistant melanoma cells of all transcriptional states.

When melanoma cells were exposed to BRAFi the presence of RANO was effective in delaying acquired resistance. The resulting VR or VR_RANO cultures showed transcriptional heterogeneity regarding the NCSC, invasive and MITF activity melanoma states as previously described^6^. However, RANO diminished the emergence of cells of the NCSC state, the NGFR-expressing melanoma state considered the most refractory not only to targeted, but also to immunotherapy^6,9^. The NCSC state has been described as similar to ‘quiescent neural stem cells’^6^, and adult neural stem cells require high levels of FAO to maintain quiescence^42^. On the other hand, glycolysis in pluripotent stem cells stimulates proliferation and inhibits differentiation^43^, and in neural crest cells it drives delamination and migration^44^. In acquired resistant VR melanoma cells of the NCSC state, differentiation dominated the biological functions, but migration and proliferation also featured. Nevertheless, gene-set enrichment analysis detected a response to lipid and fatty acid, but no function linked to glycolysis as enriched, which suggests that fatty acid metabolism maintains the NCSC state in VR cells. This would explain why cells of this melanoma state are so sensitive to RANO.

The presence of RANO enhanced the expression of the invasive state and attenuated the activity of MITF, thereby establishing cells with overlapping intermediate MITF activity and enhanced invasive state. Our data indicate that these cells use glycolysis and oxidative phosphorylation, suggesting that the exposure to RANO rewires VR cells and, while inhibiting FAO, drives glucose oxidation by promoting pyruvate entry into the tricarboxylic acid cycle. Such a situation was seen in RANO-treated rat hearts, whereby RANO increased the activity of PDH^27,45^. Along with increased oxidative phosphorylation, we detected enhanced ROS in RANO-treated VR cells, which might be the trigger for the upregulation of glutathione synthesis and consequently the activation of methionine metabolism^29^. Importantly, however, RANO also impacted on methionine metabolism by directly affecting the expression of MTAP, MAT2 and SMS in VR_RANO cells.

We found that VR_RANO cells were more sensitive to SAM and PRMT5 inhibition, which implies a RANO-induced reduction of MTAP activity, MTA accumulation and PRMT5 inhibition^33–35,46^. Indeed, the MTAP product MTR was profoundly reduced, and MTA levels were increased in VR_RANO cells. In line with this, RANO increased the expression of genes otherwise suppressed by PRMT5, including NLRC5 (ref. ^36^), the master regulator of MHC class I antigen presentation genes^37,38^. RANO also induced the expression of the type I interferons IFNA and IFNB as well as PD-L1 as previously observed in melanoma cells after PRMT5 knockdown^36^. Overall, this changed the immunogenicity of melanoma in vivo, which was demonstrated by an increase in lymphocyte infiltration, particularly of CD8^+^ cytotoxic T cells in RANO-treated tumours. This observation is in line with the fact that PRMT5 depletion specifically in melanoma cells increases T cell infiltration in vivo^36^. Yet the detected increase is intriguing, because RANO will also act on the immune microenvironment and PRMT5 depletion specifically in T cells reduces the number of peripheral T cells^47^. Moreover, PRMT5 inhibition in T cells suppresses their proliferation and IFNG production^48^. RANO on the other hand inhibits PRMT5 suppressive activities, but not only increased intra-tumoral IFNG and T cell abundance but also reduced CD8^+^ T cell exhaustion. Thus, RANO might have distinct effects, because it inhibits PRMT5 indirectly through metabolic rewiring, and this mechanism might differ from direct inhibition of PRMT5.

It is now well accepted that metabolic pathways including FAO regulate T cell differentiation and activation^20^. Fatty acid catabolism is thought to play a positive role in the antitumour immunity of CD8^+^ T cells and synergy with anti-PD-1 therapy in mice was seen with PPARA agonists^49,50^. Mechanistically, the PPARA agonist bezafibrate increased FAO, which contributed to survival, but it also upregulated oxidative phosphorylation and glycolysis, which led to enhanced naïve T cell proliferation and improved effector function of cytotoxic T cells^51^. Increased oxidative phosphorylation and lipid metabolism also correlated with increased anti-PD-1 responses in melanoma patients, which was linked to upregulation of antigen presentation and PD-L1 (ref. ^52^). Intriguingly, the latter could be mimicked in vitro using dichloroacetate, which enhances oxidative phosphorylation by indirectly activating PDH and promoting glucose oxidation. Overall, oxidative phosphorylation appears to be the Achilles’ heel to T cell proliferation and activation, and while FAO is a metabolic pathway feeding into it, so can glycolysis when pyruvate is metabolized by PDH.

In summary, we provide the scientific rationale to combine RANO with BRAFi to delay the onset of acquired resistance, and, moreover, our data suggest that the adaptation to RANO during BRAFi treatment would lead to tumours with an immunogenic phenotype that predicts improved responses to immunotherapy.

## Methods

### Cell lines and reagents

A375, WM9 and YUMM1.7 mouse melanoma cells were from the American Type Culture Collection; 501mel cells^53^ were a gift from S. Rosenberg (National Cancer Institute); 5555 cell line^54^ was a gift from R. Marais (Manchester, UK). YUMM1.7 and 5555 cells were established from male mice. FCT1 cells were established from tumours arising in a TyrCreERT2/BRAFCA/Ptenfl/+ female mouse after tamoxifen treatment. A375 and 501mel cells are from female patients, and WM9 cells are from a male patient. All cell lines had been authenticated in 2021 by STR profiling using the AmpFLSTR Identifiler Plus PCR Amplification Kit (Thermo Fisher). Cells were expanded to generate enough vials from a single batch before the start of the study. Cell lines were cultured in DMEM (61965026, Gibco) supplemented with 10% FBS (10500064, Gibco) plus 1% penicillin–streptomycin (15140122, Gibco). Cells were grown at 37 °C in a 5% CO_2_ environment. Vemurafenib (HY-12057), ETO (HY-50202A) and RANO (HY-17401) were from MedChemExpress. THIO (T9025) was from Sigma. GSK3326595 (S8664) and SAM (S5109) were from Selleckchem.

### Acquired resistance establishment protocol

Melanoma cells seeded in six-well plates were treated for 7 d with a high dose (5 or 10 µM as indicated) of vemurafenib before switching to a lower concentration of the drug (0.5 or 1 µM). Fresh medium and vemurafenib were added once weekly for a further 3–4 weeks until arising colonies grew to confluence. FAO inhibitors RANO, ETO and THIO were added once a week. For relative proliferation measurement, cells were fixed with 4% paraformaldehyde in PBS and stained with freshly prepared 0.1% crystal violet. After washing with water and drying, crystal violet was dissolved in 25 mM Tris-HCl pH 7.4, 1% SDS in water and absorbance was measured at 590 nm in a spectrophotometer.

### Colony formation assays

Cells seeded in six-well plates were treated with inhibitors or DMSO 24 h after plating. Cells were left to form colonies for 7 to 14 d (depending on the cell line) until control cells had reached an appropriate density. Then, cells were fixed and stained, and absorbance was measured as described previously^55^.

### Cell lysis and western blotting

Cells were lysed using RIPA buffer and analysed by western blotting as described^56^. Primary antibodies used were: pJAK1(Tyr1034/1035; 74129T, 1:500 dilution), pJAK1(Tyr1022/1023; 3331S, 1:500 dilution), pSTAT1(Tyr701; 7649T, 1:500 dilution), pSTAT3(Ser727; 9134T, 1:500 dilution) from Cell Signaling and ERK2 (sc-1647) from Santa Cruz Biotechnology. Detection was through enhanced chemiluminescence ECL using horseradish peroxidase-coupled secondary antibodies (1:2,000 dilution; GE Healthcare) and NOVEX ECL Chemi Substrate (Thermo Fisher).

### Metabolic flux analysis

The OCR was measured in A375VR cells using a Seahorse XFp extracellular flux analyzer (Seahorse Bioscience). In brief, A375VR cells were seeded at a density of 10,000 cells per well on a Seahorse XFp plate in DMEM medium. The next day, the medium was replaced with Seahorse XF DMEM medium pH 7.4. Then, cells were incubated for 45 min at 37 °C and 0% CO_2_ and treated with either 20 µM ETO (Merck) or RANO at the indicated concentrations during the last 15 min. Basal levels of OCR and ECAR were then recorded, followed by sequential injections of 1 µM oligomycin (Sigma), 1 µM FCCP (Sigma), 0.5 µM rotenone/antimycin A (Sigma) and 125 mM 2-deoxy-d-glucose (Sigma). OCR and ECAR data were normalized to the protein content as assessed by Bradford assay (Bio-Rad).

### Metabolomics and lipidomics analyses

#### Sample preparation

Frozen cell pellets were extracted at 2 × 10^6^ cells per ml with cold MeOH:MeCN:H_2_O (5:3:2, vol:vol:vol) or 100% methanol for metabolomics/oxylipins or lipidomics analysis, respectively. Suspensions were vortexed vigorously for 30 min at 4 °C. Insoluble material was pelleted by centrifugation (18,213*g*, 10 min, 4 °C) and supernatants were isolated for analysis by UHPLC–MS.

### Data analysis

A Vanquish UHPLC system (Thermo Fisher) was coupled to a Q Exactive mass spectrometer (Thermo Fisher) for oxylipins analysis, and an Orbitrap Exploris 120 mass spectrometer (Thermo Fisher) for metabolomics analysis. Metabolites were resolved across a 2.1 × 150 mm, 1.7-µm Kinetex SB-C18 column (Phenomenex) using a 5-min, reverse-phase gradient from a previously described method^57^. For oxylipins, the samples were analysed using a 7-min gradient across a 1.7-µm, 2.1 × 100 mm Acquity UPLC BEH column (Waters). The run order of samples was randomized and technical replicates were included to assess quality control. Raw files were converted to .mzXML files using RawConverter. The resultant files were processed with El-Maven (Elucidata) alongside the KEGG database for metabolite assignment and peak integration as previously described^58^. Lipidomics analysis used a Vanquish UHPLC system (Thermo Fisher) coupled to a Q Exactive mass spectrometer (Thermo Fisher). The samples were randomized and resolved across a 2.1 × 30 mm, 1.7-µm Kinetex C18 column (Phenomenex) using a 5-min reverse-phase gradient adapted from a previous method^59^. Technical replicates were included to assess quality control. Lipid assignments and peak integration were performed using LipidSearch v 5.0 (Thermo Fisher).

### Flow cytometry analysis

Surface and intracellular flow cytometry analyses were performed as described^60^. Cells were harvested, washed and cells immediately labelled with the indicated antibodies in a final volume of 50 μl for 10 min on ice. Cells were washed twice, resuspended in 100 μl of PBS, and analysed immediately. The following fluorochrome‐conjugated antibodies were used at 1:50 dilutions: PD-L1-APC (BioLegend, clone 29E.2.A3, ref. 329708) or B2M-FITC (BioLegend, clone 2M2, ref. 316304).

### Reactive oxygen species and glucose uptake measurements

A375VR cells were treated with 100 µM RANO for 1–4 h and ROS production was analysed by using the DCFDA/H_2_DCFDA-Cellular ROS Assay Kit (ab113851, Abcam) according to the manufacturer’s instructions. Cells treated with TBHP, a treatment that elicits ROS production, were used as positive control. A375VR and A375VR-RANO cells were treated with 100 µM RANO for 2 h, and glucose uptake was analysed by the detection of 2-deoxy-d-glucose-6-phosphate using a Glucose Uptake-Glo Assay Kit (Promega, J1341) following the manufacturer’s instructions.

### RNA analysis by qPCR

For RT–qPCR experiments in cell lines, total RNA from cells was extracted with TRIzol (Thermo Fisher). For tumour samples, fragments of tumours were homogenized in TRIzol using a Pellet Pestle Cordless Motor (Kontes). In all cases, RNA was treated with DNase (Invitrogen) and reverse transcription was performed using PrimeScript RT Reagent Kit (Takara). RT–qPCR was performed using SYBR Green (Thermo Fisher) and a QuantStudio 12K Flex qPCR system (Applied Biosystems) with triplicate biological replicates for each sample, and fold change was calculated normalized to 18S expression. A list of primer sequences is provided as Supplementary Information.

### A375 melanoma single-cell RNA-sequencing analysis

Two experimental runs were processed with the Chromium Next GEM Single Cell 3ʹ Kit v3.1 (10x Genomics), pooling all samples (parental VR, VR_RANO) per library and labelling each one of them with hashtag oligonucleotides. Chromium scRNA-seq reads were aligned to refdata-gex-GRCh38-2020-A with Cell Ranger^61^ version 4.0.0. The subsequent processing steps and analyses were performed with Seurat (v4.1.1)^62–65^. Demultiplexing samples based on data from cell ‘hashing’ was done using NormalizeData (with ‘CLR’ method) and HTODemux (positive.quantile = 0.99) Seurat functions. Doublets and negative cells were discarded for the analysis. Only cells having less than 20% mitochondrial read content, a minimum of 1,000 reads and from 100 to 20,000 detected genes were considered for downstream analyses. Ribosomal reads, which accounted for an average of 10% of the total cell-wise reads, were removed. The proportion of mitochondrial reads was regressed out during the normalization and variance stabilization of raw counts, which was performed with the sctransform method^66^. Batch effects presented by the two sequencing rounds were corrected using the IntegrateData function (normalization.method = ‘LogNormalize’) following pre-computed Anchors, which were obtained from 5,000 integration features. The first ten principal components of the integrated dataset were used to obtain the UMAP and the clustering of cells with FindClusters Seurat function (resolution of 1.2). SCT integrated counts were imputed and smoothed with magic (v.2.0.3)^67^. For every cell, gene signatures scores were calculated by taking the average magic expression of their constituent genes. FindAllMarkers was used to identify the most differentially expressed genes with parameters min.pct = 0.25 and logfc.threshold = 0.25, and considering as identity classes either Seurat clusters or treatments. For several biological functions, highly expressed cells were singled out as cells falling above the 90th percentile of the observed gene signature scores distribution across the whole dataset. Gene markers were obtained for the highly expressed populations using Seurat function FindMarkers (min.pct = 0.2, logfc.threshold = 0.2, considering only the top 200 most differentially expressed genes). These most differentially expressed sets were tested for biological enrichment with hypergeometric tests using Hallmarks, GO and KEGG as well as other previously published gene signatures. *P* values were corrected for multiple comparisons using the Benjamini–Hochberg approach. The downstream analyses detailed above (from data normalization and integration to biological enrichment) were done independently for two subsets of data: (a) parental + VR + VR_RANO cells; and (b) only VR + VR_RANO cells.

### A375 xenografts BRAFi therapy experiment

All processes involving female *Foxn1*^nu^/*Foxn1*^nu^ mice were subject to approval by the Biodonostia HRI animal experimentation ethics committee. Approval for studies with male mice was obtained from the Animal Ethics Committee of the University of Navarra (Pamplona, Spain; refs. 077-19 and 064-22) and from the Government of Navarra (experiment with male mice). These animals were housed at the animal facilities of the Center of Applied Medicine (CIMA) at conventional biosafety 2 housing conditions with environmental enrichment (ES31 2010000132, University of Navarre). When males were used, these were housed in individual cages if dominant behaviours and barbering were observed. Randomization was used to allocate mice into cages. ARRIVE guidelines were followed for animal experimentation. In total, 2 × 10^6^ A375 human melanoma cells were injected into both flanks of nude mice (8 weeks of age). External callipers were used to measure tumour volume. Once tumours reached the size of ~60 mm^3^, mice were assigned to different groups (*n* = 7 per group). Drugs or vehicle were administered by intraperitoneal injection. Vehicle, vemurafenib (25 mg per kg body weight), RANO (50 mg per kg body weight) or a combination was administered once daily for up to 50 d and tumour volumes were measured every 3 d. Tumours were collected and snap frozen for RNA extraction.

### Mouse 5555 anti-PD-L1 therapy experiment

All experiments involving animals were performed in accordance with the European Community Council Directive (2010/63/EU) and Spanish legislation. The protocols were approved by the CSIC Ethics Committee as well as the Animal Welfare Committee at the Instituto de Neurociencias CSIC-UMH (Alicante, Spain). Mice were hosted in a pathogen-free facility under controlled temperature, humidity, ad-libitum feeding and 12-h light–dark cycle. All experiments were performed in 7- to 8-week-old mice C57BL/6J purchased from Charles River (Jackson). Mice were injected subcutaneously with 5 × 10^6^ mouse melanoma cells from the 5555 cell line^54^ into the right flank on day 0. Animals were monitored daily until a tumour was evident and palpable (70 mm^3^), around day 6 after injection (6DPI). Animals were then divided into groups and treated accordingly: The RANO group was given a 150 mg per kg body weight by oral gavage of RANO (MedChemExpress, HY-17401) on 6DPI and 300 mg per kg body weight from 7DPI to the endpoint. The PD-L1 group was given 10 mg per kg body weight anti-PD-L1 via intraperitoneal injection (BioxCell, PD-L1 B7-H1, BE0101) starting on 6DPI and every 3 d for a total of five doses after which mice were kept until the relevant endpoint. The PD-L1 + RANO group was given both treatments as described above. The control group was injected with PBS daily until the endpoint. Mice were euthanized before the tumour volume reached 1,500 mm^3^ and culled humanely. Tumour size was measured every 3 d starting on 6DPI and was determined by calliper measurements of tumour length, width and depth, and volume was calculated as ‘volume = 0.5236 × length × width × depth (mm)’. Following tumour resection, the samples were divided (whenever possible given the tumour size) for whole-tissue snap freezing for RNA extraction and formalin embedded for histology.

### Tumour stroma single-cell RNA-sequencing analysis

scRNA-seq samples were obtained from 5555 mouse melanomas at the specific sample group endpoint described. Tumours were processed into single-cell suspensions following this protocol. First, animals were culled, and the tumour resected from the skin. A piece of 100 mg was weighed and chopped using sharp razors and scalpels in cold DMEM without FBS and penicillin–streptomycin on ice. A final concentration of 0.25 mg ml^−1^ of Liberase DH (Merck, 54010544001), 0.55 mg ml^−1^ of Dispase II (Merck, 4942078001) and 150 U ml^−1^ of DNAse (Roche, 11284932001) was added in 1 ml of solution. Samples were incubated at 37 °C for 30 min and then homogenized with increasing gauge syringes up to 25 G. Then, samples were centrifuged at 300*g* 4 °C for 5 min and 1× red-blood lysis buffer (BioLegend, 420301) was applied for 5 min at room temperature (RT) and then filtered through a 40-µm cell strainer. Samples were centrifuged again for at 300*g* 5 min at 4 °C, resuspended in FACS buffer (sterile-filtered 1% FCS, 2 mM EDTA, 25 mM HEPES in 1× PBS) and incubated with TruStain FcX mouse antibody (BioLegend, 156604; 1:50 dilution) for 10 min on ice in darkness. This was followed by incubation with anti-CD45-PE (BioLegend, 103106; 1:100 dilution) and 1 µg ml^−1^ DAPI for 30 min on ice and darkness. Finally, samples were washed twice with FACS buffer and centrifuged at 300*g* at 4 °C for 5 min.

FACS isolation was performed by gating a DAPI-negative population (live cells) and by double discrimination of singles and doublets. Then CD45-PE^+^ cells were sorted into individual tubes per group condition, with 15,000 cells per group. Cell integrity and number were assessed by Trypan blue and bright-field microscopy in every condition. Live CD45-PE^+^ cells were encapsulated into droplets and libraries were prepared using Chromium Single Cell Reagents kit v3.1 according to the manufacturer’s protocol (10x genomics, PN-1000269). The generated libraries were sequenced using an Illumina NovaSeq 6000 sequencer to obtain approximately 40,000 reads per cell.

Chromium scRNA-seq reads were aligned to refdata-cellranger-mm10-3.0.0 with Cell Ranger^61^ version 4.0.0. The subsequent processing steps and analyses were performed with Seurat R package version (v4.1.1)^62–65^. Only cells having <20% mitochondrial read content, and a minimum of 2,000 reads were considered for downstream analyses. Ribosomal reads, which accounted for an average of 19% of the total cell-wise reads, were removed. The proportion of mitochondrial reads was regressed out during the normalization and variance stabilization of raw counts, which was performed with the ‘sctransform’ method^66^. The first 15 principal components of the SCT dataset were used to obtain the UMAP and the clustering of cells with the ‘FindClusters’ Seurat function (resolution of 1.2). Seurat clusters were annotated based on expressions of gene markers of particular cell types using the datasets included in the packages of Enrichr^68^ and Panglao DB^69^.

### Immunofluorescence and imaging

Tissues were fixed in 4% paraformaldehyde overnight and washed three times with PBS. Subsequently, tissues were treated with increasing concentrations of sucrose up to 30% until the tissues sank to the bottom of the tubes. The tissues were then embedded in optimal cutting temperature (OCT) compound (Tissue-Tek, 127217) and stored at −80 °C until further processing. OCT blocks were cut into 8-μm sections using a cryotome (Leica CM1860 UV) and mounted onto Superfrost glass slides (12-550-15). Immunofluorescence staining was performed on OCT sections. To this end, samples were blocked with a solution containing 3% BSA, 1% normal goat serum and 0.1% Tween 20 for 1 h at RT. Primary antibody incubation was performed overnight at 4 °C with a solution containing 1% BSA and 0.1% Tween 20. After extensive washing with 1× PBS containing 0.1% Tween 20, the sections were incubated with the secondary antibodies and DAPI in a solution containing 1% BSA and 0.1% Tween 20 for 1 h at RT. After washing the secondary antibodies, the sections were mounted in Dako Fluorescence Mounting Medium (Thermo Fisher, S3023). The primary antibodies used were CD8a-FITC (BioLegend, ref. 100705) and CD335-FITC (BioLegend, ref. 137605) at a 1:50 dilution. The secondary antibody used was AF488 anti-Rat (Thermo Fisher, A11006) at a 1:1,000 dilution.

For high-resolution imaging, a confocal LSM880-Airyscan (Zeiss) with a ×25 oil objective and the Airyscan super-resolution imaging technique were used. For image quantification, whole tissues were imaged using the Axioscan 7 (Zeiss) with a ×20 objective. Whole-tissue images were processed using Arivis Vision 4D software (Zeiss). A pipeline was created to detect DAPI and antibody double-positive cells, and all the tissues were processed and analysed for further quantification.

### Data analysis and statistics

GraphPad Prism version 7.00 for Mac OS (GraphPad Software) was used for analysis. One-way ANOVA or Student’s *t*-test was used for bar graph analyses, log-rank test for Kaplan–Meier survival analyses, Pearson correlation for coexpression analyses and two-way ANOVA (mixed-model) analysis for tumour growth. Data represent the results for assays performed from at least three replicates, and values are the mean ± s.e.m. *P < 0.05; **P < 0.01; ***P < 0.001.

### Reporting summary

Further information on research design is available in the Nature Portfolio Reporting Summary linked to this article.

### Supplementary information

Supplementary Information Supplementary Tables 1 and 2 and Supplementary Fig. 1.

Reporting Summary

### Source data

Source Data Fig. 1 Data from CFAs and qRt-PCR.

Source Data Fig. 2 Data from CFAs, gene expression measurements, ECAR and metabolomics.

Source Data Fig. 3 Data from tumour volume measurements and gene expression measurements.

Source Data Fig. 6 Data from ROS levels measurements and glucose uptake measurements.

Source Data Fig. 7 Data from gene expression measurements and metabolomics.

Source Data Fig. 7 Unprocessed western blots from Fig. 7.

Source Data Fig. 8 Data from tumour volume measurements and gene expression measurements.

Source Data Extended Data Fig. 1 Data from gene expression measurements.

Source Data Extended Data Fig. 2 Data from colony formation assays, gene expression measurements, ECAR and metabolomics.

Source Data Extended Data Fig. 3 Data from gene expression measurements.

Source Data Extended Data Fig. 4 Data from tumour volume measurements and gene expression measurements.

Source Data Extended Data Fig. 7 Data from tumour volume measurements.

Source Data Extended Data Fig. 8 Data from gene expression measurements.

## Data Availability

scRNA-seq data of human melanoma cell lines are deposited in ArrayExpress under accession number E-MTAB-12412. scRNA-seq data of mouse melanoma tumours are deposited in ArrayExpress under accession number E-MTAB-12315. Lipidomic data of human cell lines are deposited in Metabolomics Workbench under accession number ST002713. Metabolomic data of human cell lines are deposited in Metabolomics Workbench under accession number ST002712. No specific code was created to analyse the data, but details of code used in this study can be obtained if required. Source data are provided with this paper.

## Code availability

Code used for human melanoma cell scRNA-seq analysis is available at: 10.5281/zenodo.8093417. Code used for mouse melanoma scRNA-seq analysis is available at: 10.5281/zenodo.8093458.
