# Supplementary material for: Metabolic rewiring induced by ranolazine improves melanoma responses to targeted therapy and immunotherapy
Source: Nat Metab. 2023 Aug 10;5(9):1544–62. doi: 10.1038/s42255-023-00861-4 (PMC10513932; doi:10.1038/s42255-023-00861-4)
Supplement: Supplementary file 1 — Supplementary Tables 1 and 2 and Supplementary Fig. 1. [file 42255_2023_861_MOESM1_ESM.pdf]

nature metabolism

Article https://doi.org/10.1038/s42255-023-00861-4

Metabolic rewiring induced by ranolazine improves melanoma responses to targeted therapy and immunotherapy

In the format provided by the authors and unedited

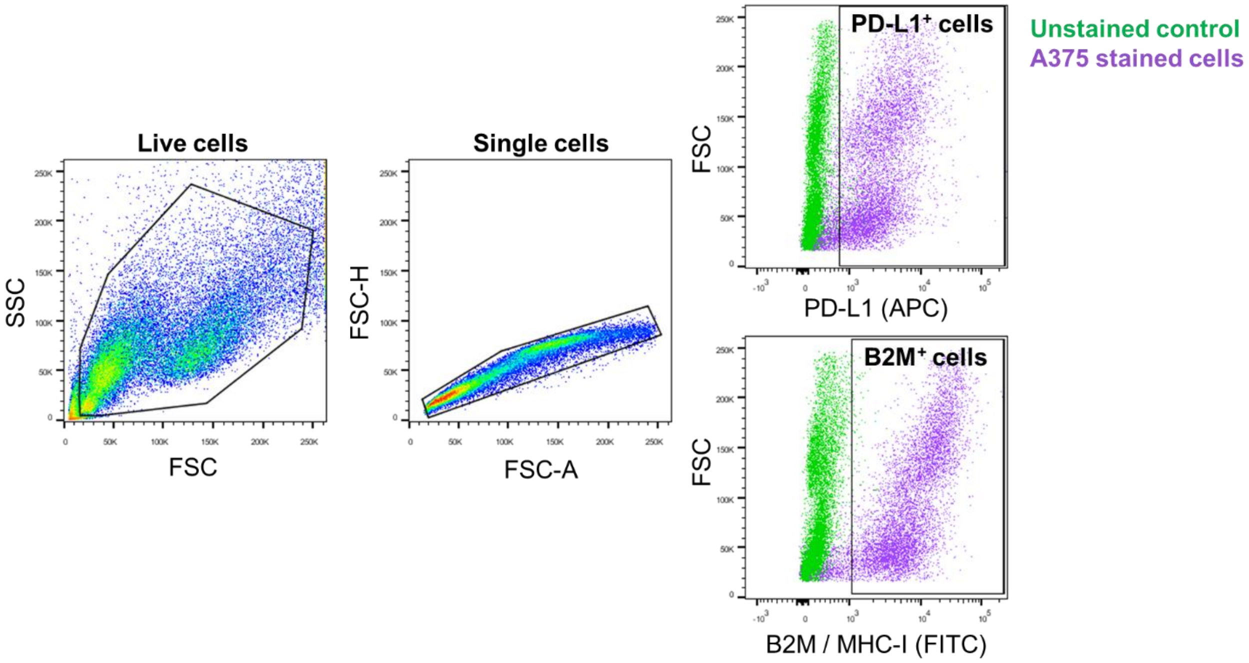

Supplemental Fig 1. Gating strategy followed to analyse FACS data. A375-VR and A375-VR-RAN cells were cultured for 24 hours, after which cells were harvested, blocked with Fc blocking buffer and stained with fluorochrome-conjugated antibodies. Samples were acquired with a FACS Canto II Flow cytometer. Data were exported as FCS3.0 files and analysed using FlowJo software. Dead cells and doublets were excluded. PD-L1^{+} and B2M^{+} cells were gated according to unstained controls. Mean fluorescence intensity (MFI) of the APC and FITC channels was analyzed using FlowJo's statistic analysis tool.

Homo Sapiens		
Gene	Forward sequence (5'-3')	Reverse sequence (5'-3')
DUSP6	CCGCAGGAGCTATACGAGTC	CGTAGAGCACCACTGTGTCG
MITF	CCGTCTCTCACTGGATTGGT	TACTTGGTGGGGTTTTCGAG
AXL	AACCTTCAACTCCTGCCTTCTCG	CAGCTTCTCCTTCAGCTCTTCAC
CPT1A	GACGTGGGAAAAATAAGCAGTC	ACATCGGCCGTGTAGTAGAGAT
CD36	TCCACAGGAAGTGATGATGAAC	TGCAGGAAAGAGACTGTGTTGT
PPARGC1A	CAGGCTGGCAGTGTGCTG	CTGCACCACTTGAGTCCACC
PPARA	GTCACACAACGCTATCCGTTT	AGGCATTGTAGATGTGCTTGG
ACOX1	GGTTTAAAAATTTTGTGCACCGAGG	CGAAGGTGAGTTCCATGACCC
ACADS	CACGCCTTTCACCAGTGGTGAC	GGCATTGGTGATCCAGGCTTTG
EHHADH	ATAGGATTGCCCACGCAGAG	TGCTAAAATACGTCTTCCTGAGGT
GLUT3	TCCACGCTCATGACTGTTTC	GCCTGGTCCAATTTCAAAGA
HK2	GAGTTTGACCTGGATGTGGTTGC	CCTCCATGTAGCAGGCATTGCT
NLRC5	AGTGGCTCTTCCGCTTGGACAT	CGGAACCCTAAGAACTTGGCTG
TAP1	GCAGTCAACTCCTGGACCACTA	CAAGGTTCCCACTGCTTACAGC
B2M	CCACTGAAAAAGATGAGTATGCCT	CCAATCCAAATGCGGCATCTTCA
PSMB9	CGAGAGGACTTGTCTGCACATC	CACCAATGGCAAAAGGCTGTCG
IFNA	AGAAGGCTCCAGCCATCTCTGT	TGCTGGTAGAGTTCGGTGCAGA
IFNB	CTTGGATTCCTACAAAGAAGCAGC	TCCTCCTTCTGGAACTGCTGCA
IFNG	TGATGGCTGAACTGTCGCCAGC	ACTGGGATGCTCTTCGACCTCG
PD-L1	TGCCGACTACAAGCGAATTACTG	CTGCTTGTCCAGATGACTTCGG
18S	GCAATTATTCCCCATGAACG	GGGACTTAATCAACGCAAGC
GLUT2	CACACAAGACCTGGAATTGACA	CGGTCATCCAGTGGAACAC
GLUT4	CTGGGCCTCACAGTGCTAC	GTCAGGCGCTTCAGACTCTT
HK1	CTGCTGGTGAAAATCCGTAGTGG	GTCCAAGAAGTCAGAGATGCAGG
GPI	CTGGTAGACGGCAAGGATGTGA	TCCGTGATGGTCTTGCCTGTGT
PFK	AAGAAGTAGGCTGGCACGACGT	GCGGATGTTCTCCACAATGGAC
ALDOA	GACACTCTACCAGAAGGCGGAT	GGTGGTAGTCTCGCCATTTGTC
ENO1	AGTCAACCAGATTGGCTCCGTG	CACAACCAGGTCAGCGATGAAG
PKM2	ATGGCTGACACATTCCTGGAGC	CCTTCAACGTCTCCACTGATCG
PGM1	TGATGGACGCGAGCAAACTGTC	ATGTCCTCCACACTCTGCTTGC
PGK1	CCGCTTTCATGTGGAGGAAGAAG	CTCTGTGAGCAGTGCCAAAAGC
SCN5A	TTGCTTGTTATGGTCATTGGC	GTTGTTCATCTCTCTATTGGC
SCN9A_1	GATGATCGGCGGGCTAGGTTGC	GAGGTCCTGGGGGAGGCAACAT
SCN10A_1	AGGGGATCCGCACACTGCTCTT	ATGCCAGCCTCCCACCTCACAT
CYP3A4	CCGAGTGGATTTCCTTCAGCTG	TGCTCGTGGTTTCATAGCCAGC
CYP3A5	ACGGTCATTGCTGTCTCCAACC	GTGACAGGCTTGCCTTTCTCTG
CYP2D6	GCAAGAAGTCGCTGGAGCAGTG	CTCACGGCTTTGTCCAAGAGAC
**Mus musculus**		
**Gene**	**Forward sequence (5'-3')**	**Reverse sequence (5'-3')**
Pd-l1	TGCGGACTACAAGCGAATCACG	CTCAGCTTCTGGATAACCCTCG
Ifnb1	GCCTTTGCCATCCAAGAGATGC	ACACTGTCTGCTGGTGGAGTTC
Ifna1	GGATGTGACCTTCCTCAGACTC x	ACCTTCTCCTGCGGGAATCCAA
Ifng	CAGCAACAGCAAGGCGAAAAAGG	TTTCCGCTTCCTGAGGCTGGAT
Nlrc5	ACCAATGCACGTCTCCGGGGTA	TGGGTTATGGTGGGCAGGAGGG
Tap1	GACTCCTTGCTCTCCACTCAGT	AACGCTGTCACCGTTCCAGGAT
B2m	ACAGTTCCACCCGCCTCACATT	TAGAAAGACCAGTCCTTGCTGAAG
Psmb9	TACCGTGAGGACTTGTTAGCGC	GGCTGTCGAATTAGCATCCCTC
Cd4	GAGTTCCCAGAAGAAGATCAC	AAGGCGAACCTCCTCTAA
Cd8	CCATGAGGGACACGAATAATAA	GAGTTCACTTTCTGAAGGACTG
Cd3g	CAGTCAAGAGCTTCAGACAAG	GATGGCTGTACTGGTCATATTC
Cd45	CCCTTCTTCTGCCTCAAAGT	GTGGATAACACACCTGGATGAT
18S	CGCCGCTAGAGGTGAAATTC	TCTTGGCAAATGCTTTCGC

Supplementary Table 1: Primers´ sequence used for mRNA expression assays

Gene	Reference
Pdcd1	https://www.ncbi.nlm.nih.gov/pmc/articles/PMC5731479/
Ctla4	https://pubmed.ncbi.nlm.nih.gov/11244047/
Havcr2	https://clinicalepigeneticsjournal.biomedcentral.com/articles/10.1186/s13148-019-0752-8
Lag3	https://www.ncbi.nlm.nih.gov/pmc/articles/PMC5617435/
Btla	https://aacrjournals.org/cancerres/article/72/4/887/577961/CD8-T-Cells-Specific-for-Tumor-Antigens-Can-Be
Cd244	https://www.ncbi.nlm.nih.gov/pmc/articles/PMC3104769/
Cd160	https://pubmed.ncbi.nlm.nih.gov/25255144/
Entpd1	https://www.nature.com/articles/s43856-022-00163-y
Tigit	https://journals.aai.org/jimmunol/article/186/3/1338/84916/Cutting-Edge-TIGIT-Has-T-Cell-Intrinsic-Inhibitory
Eomes	https://www.frontiersin.org/articles/10.3389/fimmu.2018.02981/full
Tim3	https://www.pnas.org/doi/full/10.1073/pnas.1009731107

Supplementary Table 2: List of genes from the Exhaustion Gene Signature
