# Supplementary material for: Metabolic rewiring induced by ranolazine improves melanoma responses to targeted therapy and immunotherapy
Source: Nat Metab. 2023 Aug 10;5(9):1544–62. doi: 10.1038/s42255-023-00861-4 (PMC10513932; doi:10.1038/s42255-023-00861-4)
Supplement: Supplementary file 2 — Reporting Summary [file 42255_2023_861_MOESM2_ESM.pdf]

nature portfolio

Corresponding author(s): Imanol Arozarena; Berta Sanchez-Laorden, Salvador Aznar Benitah

Last updated by author(s): May 9, 2023

Reporting Summary

Nature Portfolio wishes to improve the reproducibility of the work that we publish. This form provides structure for consistency and transparency in reporting. For further information on Nature Portfolio policies, see our Editorial Policies and the Editorial Policy Checklist.

Statistics

For all statistical analyses, confirm that the following items are present in the figure legend, table legend, main text, or Methods section.

n/a	Confirmed	
☒	☐	The exact sample size (*n*) for each experimental group/condition, given as a discrete number and unit of measurement
☒	☐	A statement on whether measurements were taken from distinct samples or whether the same sample was measured repeatedly
☒	☐	The statistical test(s) used AND whether they are one- or two-sided<br>*Only common tests should be described solely by name; describe more complex techniques in the Methods section.*
☒	☐	A description of all covariates tested
☒	☐	A description of any assumptions or corrections, such as tests of normality and adjustment for multiple comparisons
☒	☐	A full description of the statistical parameters including central tendency (e.g. means) or other basic estimates (e.g. regression coefficient) AND variation (e.g. standard deviation) or associated estimates of uncertainty (e.g. confidence intervals)
☒	☐	For null hypothesis testing, the test statistic (e.g. *F*, *t*, *r*) with confidence intervals, effect sizes, degrees of freedom and *P* value noted<br>*Give P values as exact values whenever suitable.*
☒	☐	For Bayesian analysis, information on the choice of priors and Markov chain Monte Carlo settings
☒	☐	For hierarchical and complex designs, identification of the appropriate level for tests and full reporting of outcomes
☐	☒	Estimates of effect sizes (e.g. Cohen's *d*, Pearson's *r*), indicating how they were calculated

Our web collection on statistics for biologists contains articles on many of the points above.

Software and code

Policy information about availability of computer code

Data collection	No software was used for data collection
Data analysis	Graphpad Prism 7, R 4.1. 3, Cell Ranger 4.0.0, Seurat R package 4.1.1, El-Maven (Elucidata) v 0.12.0, LipidSearch v 5.0 (Thermo), FlowJo (BD) v 10.7.1, Arivis 4D software (Zeiss) and Zen Blue 3.8 (Zeiss)<br><br>Code used for human melanoma cell scRNAseq analysis is available at:<br>https://doi.org/10.5281/zenodo.8093417<br><br>Code used for mouse melanoma scRNAseq analysis is available at:<br>https://doi.org/10.5281/zenodo.8093458

For manuscripts utilizing custom algorithms or software that are central to the research but not yet described in published literature, software must be made available to editors and reviewers. We strongly encourage code deposition in a community repository (e.g. GitHub). See the Nature Portfolio guidelines for submitting code & software for further information.

Data

Policy information about availability of data

All manuscripts must include a data availability statement. This statement should provide the following information, where applicable:

- Accession codes, unique identifiers, or web links for publicly available datasets
- A description of any restrictions on data availability
- For clinical datasets or third party data, please ensure that the statement adheres to our policy

scRNAseq data of human melanoma cell lines are deposited in ArrayExpress under accession number E-MTAB-12412.
scRNAseq data of mouse melanoma tumours are deposited in ArrayExpress under accession number E-MTAB-12315.
Lipidomic data of human cell lines are deposited in Metabolomics Workbench under accession number Study ST002713
Metabolomic data of human cell lines are deposited in Metabolomics Workbench under accession number Study ST002712.
No specific code was created to analyze the data, but details of code used in this study can be obtained if required.

Research involving human participants, their data, or biological material

Policy information about studies with human participants or human data. See also policy information about sex, gender (identity/presentation), and sexual orientation and race, ethnicity and racism.

Reporting on sex and gender	This study does not include Human research participants
Reporting on race, ethnicity, or other socially relevant groupings	This study does not include Human research participants
Population characteristics	This study does not include Human research participants
Recruitment	This study does not include Human research participants
Ethics oversight	This study does not include Human research participants

Note that full information on the approval of the study protocol must also be provided in the manuscript.

Field-specific reporting

Please select the one below that is the best fit for your research. If you are not sure, read the appropriate sections before making your selection.

☒ Life sciences ☐ Behavioural & social sciences ☐ Ecological, evolutionary & environmental sciences

For a reference copy of the document with all sections, see nature.com/documents/nr-reporting-summary-flat.pdf

Life sciences study design

All studies must disclose on these points even when the disclosure is negative.

Sample size	Sample size was selected following the recommendations from the "Organo Evaluador de Proyectos" (University Miguel Hernandez) to calculate the size of the cohorts and following the requirements for the principles of the "3Rs" (reduction, refinement and replacement). The sample size was calculated using previous information regarding this type of biological assay, tumour syngeneic subcutaneous injections. Given known group means and the standard deviation of the mean between both groups, the sample size was assigned given the assumption of alpha = 0,05 and power = 0,80. Given this information, the number of animals per group should be equal or above 5 animals.
Data exclusions	For figure 8c, g, h and i and extended data figure 6c-h, several animals were excluded from the study given the following conditions. For sub-cutaneous tumour cell injections, after initial monitoring, around day 6 post injection (6DPI), animal tumours were measured with caliper and outliers were identified by using a ROUT method for outlier identification with Q=1%. Animals that did not present visible tumours were discarded and animals with a volume size above the media and considered outliers were discarded as well. These animal exclusions were given on the basis that the nature of the study is to evaluate the tumour growth and evolution. If some of the individuals would present too large tumours in already exponential growth, the data and the experiment would be confounded.
Replication	Biological and technical replicates are detailed in the figure legends and method section. Most experiments required no less than 3 replicates (technical and biological). Single cell sequencing (10X) of cell lines include duplicates.
Randomization	Mice have specific codes given by the software Anibio v6.35 and randomized to established treament groups
Blinding	For figure 3c-d, Extended Data Figure 3 c-h, Figure 8,g -i, and Extended data Fifure 6 c-g, given the randomised groups, the data was kept as it was given originally; the mice number and group cage. The person obtaining the samples and later on analysing the results did not have access to the biological data until the analysis was performed.

Reporting for specific materials, systems and methods

We require information from authors about some types of materials, experimental systems and methods used in many studies. Here, indicate whether each material, system or method listed is relevant to your study. If you are not sure if a list item applies to your research, read the appropriate section before selecting a response.

Materials & experimental systems

n/a	Involved in the study
☐	☒ Antibodies
☐	☒ Eukaryotic cell lines
☒	☐ Palaeontology and archaeology
☐	☒ Animals and other organisms
☐	☒ Clinical data
☒	☐ Dual use research of concern
☒	☐ Plants

Methods

n/a	Involved in the study
☒	☐ ChIP-seq
☐	☒ Flow cytometry
☒	☐ MRI-based neuroimaging

Antibodies

Antibodies used

-The anti-mouse PDL1(B7-H1) from Bioxcell clone 10F.9G2 was used for mice experiments with immunotherapy. Dilution: 10mg/kg.
CD45-APC clone 30-F11 was used (Ref. 103112. Lot: 8320220, Biolegend.). This antibody was quality tested for Flow Cytometry from the supplier and we also have tested via titration of the antibody in the current sample (1:100).
-pJAK1 (Tyr1034/1035): recognizes endogenous levels of Jak1 protein only when phosphorylated at Tyr1034/1035. This antibody also detects Jak1 when phosphorylated at just Tyr1034. Supplier: Cell Signaling; Catalog number: 74129T; Clone D7N4Z. Lot number 1. Dilution: 1:500
-pJAK1 (Tyr1022/1023): detects endogenous levels of Jak1 only when phosphorylated at tyrosines 1022/1023. Human Jak1 residues Tyr1034 and Tyr1035 historically have been referenced as Tyr1022 and Tyr1023. This antibody may cross-react with phospho-Jak2. Supplier: Cell Signaling; Catalog number: 3331S. Dilution: 1:500.
-pSTAT1 (Tyr701): recognizes endogenous levels of Stat1 protein only when phosphorylated at Tyr701. Supplier: Cell Signaling; Catalog number: 7649T; Clone D4A7. Lot number: 5. Dilution: 1:500.
-pSTAT3 (Ser727): recognizes endogenous levels of Stat3 only when phosphorylated at Ser727. The antibody does not significantly cross-react with the corresponding phospho-serines of other Stat proteins. Supplier: Cell Signaling; Catalog number: 9134T; Lot number: 21. Dilution: 1:500
-ERK2: specific for an epitope mapping between amino acids 345-358 at the C-terminus of ERK 2 of human origin. Supplier: Santa Cruz Biotechnology; Catalog number: sc-1647; Lot number: A2219. Dilution: 1:1000.
- Anti-Rabbit IgG, HRP-linked polyclonal antibody: VWR-GE Healthcare, Ref. NA934, host: Donkey, Lot: 17348043. Dilution: 1:2000
- Anti-mouse IgG, HRP-linked polyclonal antibody : VWR-GE Healthcare, Ref. NA931, host Sheep. Lot: 17376630. Dilution: 1:2000
-APC anti-human CD274 (B7-H1, PD-L1) Antibody: Supplier: Biolegend Clone 29.E2.A3; Ref: 329708. Lot: B330509. Dilution: 1:50.
-FITC anti-human β2-microglobulin Antibody. Supplier: Biolegend; Clone: 2M2; Ref: 316304. Lot: B261327. Dilution: 1:50.
-Antibody CD8a-FITC was from Biolegend; Ref. 100706. Dilution: 1:50. Lot: B312822
-Antibody CD335-FITC was from Biolegend; Ref: 137606. Dilution: 1:50. Lot: B355931
- Secondary antibody AF488 anti-Rat from Thermo Fisher, Ref:11006. Dilution: 1:1000.

Validation

-PD-L1(B7-H1) clone 10F.9G2 raised against mouse PD-L1, also known as B7_H1 or CD274. This antibody was validated in the supplier company confirming that this clone binds to its target antigen by using a C-tag terminus PD-L1 antibody. Other references have been published using this source of antibody. Furthermore we also validated this antibody in-vivo in a small number of animals in a pilot study for this project.

All antibodies against phosphorylated forms of JAK1, STAT1 or STAT3 or against ERK2 were usedfor western-blot . They were purchased after consulting the literature. Final working conditions were decided after testing several dilutions (lower and higher than those instructed by the manufacturer).
-pJAK1 (Tyr1034/1035): Rabbit monoclonal antibody for Western Blot. Produced by immunizing animals with a synthetic phospho-peptide corresponding to residues surrounding Tyr1034/1035 of human Jak1 protein.
-pJAK1 (Tyr1022/1023): Rabbit polyclonal antibody for Western Blot. Polyclonal antibodies are produced by immunizing animals with a synthetic phosphopeptide corresponding to residues surrounding Tyr1034/1035 of human Jak1. Antibodies are purified by protein A and peptide affinity chromatography.
-pSTAT1 (Tyr701): Rabbit monoclonal antibody for Western Blot. Monoclonal antibody is produced by immunizing animals with a synthetic peptide corresponding to residues surrounding Tyr701 of human Stat1 protein.
-pSTAT3 (Ser727): Rabbit polyclonal antibody for Western Blot. Polyclonal antibodies are produced by immunizing animals with a synthetic phosphopeptide corresponding to residues surrounding Ser727 of mouse Stat3. Antibodies are purified by protein A and peptide affinity chromatography.
-ERK2: Mouse monoclonal IgG2b κ for Western Blot.Primary antibodies like Anti-ERK 2 Antibody (D-2) for mammalian target proteins are recommended for the detection of a range of mammalian species, primarily of mouse, rat and human species.

-APC anti-human CD274 (B7-H1, PD-L1) Antibody: Inmunogen: Full length human PD-L1 . The antibody was purified by affinity chromatography, and conjugated with APC under optimal conditions. We tested this antibody by using different dilutions of the suggested concentration (2 μg per 106 cells) and stick with the suggested concentration.
-FITC anti-human β2-microglobulin Antibody. Immunogen. Purified human β2-microglobulin. The antibody was purified by affinity chromatography, and conjugated with FITC under optimal conditions. We tested this antibody by using different dilutions of the

suggested concentration (1 µg per 106 cells) and stick with the suggested concentration.

-For the scRNAseq from mouse tumours, the CD45-APC monoclonal antibody raised against mouse CD45 (Ref. 103112 Biolegend) was quality tested for Flow Cytometry from the supplier. This lot has been QC tested by immunofluorescent staining with flow cytometric analysis by using 0.25 µg per 106 cells using mouse splenocytes. We tested this antibody by using different dilutions of the suggested concentration (0.25 µg per 106 cells) and stick with the suggested concentration.

-CD8A-FITC, a monoclonal antibody against mouse CD8A (Ref. 100706. Lot: B312822 Biolegend). This lot has been QC tested by immunofluorescent staining with flow cytometric analysis by using 0.25 µg per 106 cells using mouse splenocytes. We tested this antibody by using different dilutions of the suggested concentration (0.25 µg per 106 cells) and stick with the suggested concentration.
-CD335-FITC, a monoclonal antibody against mouse CD335/NK1.1.(Ref. 137606. Lot: B355931. Biolegend). This lot has been QC tested by immunofluorescent staining with flow cytometric analysis by using 0.25 µg per 106 cells using mouse splenocytes. We tested this antibody by using different dilutions of the suggested concentration (0.25 µg per 106 cells) and stick with the suggested concentration
These antibodies were quality tested for Flow cytometry and we have validated them for immunofluorescence.

Eukaryotic cell lines

Policy information about cell lines and Sex and Gender in Research

Cell line source(s)	A375, WM9 and 501mel human melanoma cells and YUMM1.7 mouse melanoma cells were from ATCC; 5555 cells 49 were a kind gift from Richard Marais (Manchester, UK). Yumm1.7 and 5555 cells where established from male mice. FCT1 cells were established from tumours arising in a TyrCreERT2/BRAFCA/Ptenfl/+ female mouse upon tamoxifen treatment. A375 and 501mel cells are from female patients, Wm9 cells are from a male patient.
Authentication	All cell lines had been authenticated in 2021 by STR profiling using the AmpFLSTR® Identifiler® Plus PCR Amplification Kit (Thermofisher)
Mycoplasma contamination	All cell lines were mycoplasma contamination tested with negative results. Cell pellets were boiled in water to generate cell lysates. Mycoplasma detection was performed in all cell lysates by PCR using these primers: forward sequence (5'-3') GGGAGCAAACAGGATTAGATACCCT; Reverse sequence (5'-3') TGCACCATCTGTCACTCTGTTAACCTC. Amplification product was detected at 270bp in 2% agarose gel.
Commonly misidentified lines (See ICLAC register)	No commonly misisdentified cell lines were used.

Animals and other research organisms

Policy information about studies involving animals; ARRIVE guidelines recommended for reporting animal research, and Sex and Gender in Research

Laboratory animals	For the subcutaneous injections of 5555 melanoma cell line, C57BL/6 male mice were used. The age of the animals was between 7-8 weeks average.<br>For the subcutaneous injections of A375 melanoma cells Foxn1nu/Foxn1nu nude animals mice were used. The age of the animals was 8 weeks average.<br>The temperature of the anial facility was kept between 20 and 24ºC and humidity was maintained at 50% -/+10%.
Wild animals	No wild animals were used in the study.
Reporting on sex	For the subcutaneous injections of 5555 melanoma cell lines, all C57BL/6 animales used were males and females<br>For the subcutaneous injections of A375 human melanoma cell, all Foxn1nu/Foxn1nu nude animals used were females and males
Field-collected samples	No field collected samples were used in the study.
Ethics oversight	All experiments involving animals were performed in accordance with the European Community Council Directive (2010/63/EU) and Spanish legislation. The protocols were approved by the CSIC Ethical Committee and the Animal Welfare Committee at the Instituto de Neurociencias CSIC-UMH, Alicante, Spain. Mice were hosted in a pathogen-free facility under controlled temperature, humidity, ad-libitum feeding and 12hour light/dark cycle.<br>All processes involving Foxn1nu/Foxn1nu nude mice animals were subject to approval by the Biodonostia HRI animal experimentation ethics committee (experment with females) and the Centro de Investigacion Medica Aplcada in Pamplona (experiments with A375 cells in male mice).

Note that full information on the approval of the study protocol must also be provided in the manuscript.

Clinical data

Policy information about clinical studies
All manuscripts should comply with the ICMJE guidelines for publication of clinical research and a completed CONSORT checklist must be included with all submissions.

Clinical trial registration	*Provide the trial registration number from ClinicalTrials.gov or an equivalent agency.*

Study protocol	*Note where the full trial protocol can be accessed OR if not available, explain why.*
Data collection	*Describe the settings and locales of data collection, noting the time periods of recruitment and data collection.*
Outcomes	*Describe how you pre-defined primary and secondary outcome measures and how you assessed these measures.*

Flow Cytometry

Plots

Confirm that:

☒ The axis labels state the marker and fluorochrome used (e.g. CD4-FITC).

☒ The axis scales are clearly visible. Include numbers along axes only for bottom left plot of group (a 'group' is an analysis of identical markers).

☒ All plots are contour plots with outliers or pseudocolor plots.

☒ A numerical value for number of cells or percentage (with statistics) is provided.

Methodology

Sample preparation	Cell from established cell lines were harvested and washed in PBS, before directly being stained with anti:PD-L1 or B2M antibodies. After some washing steps samples were ready to be analized
Instrument	BD FACSCanto II
Software	FlowJo v 10.7.1
Cell population abundance	Samples are established cell lines, no other populations present
Gating strategy	Single cells were gated and PD-L1 or B2M staining was detected in that population

☒ Tick this box to confirm that a figure exemplifying the gating strategy is provided in the Supplementary Information.
