# Supplementary figures and images for: Metabolic rewiring induced by ranolazine improves melanoma responses to targeted therapy and immunotherapy
Source: Nat Metab. 2023 Aug 10;5(9):1544–62. doi: 10.1038/s42255-023-00861-4 (PMC10513932; doi:10.1038/s42255-023-00861-4)

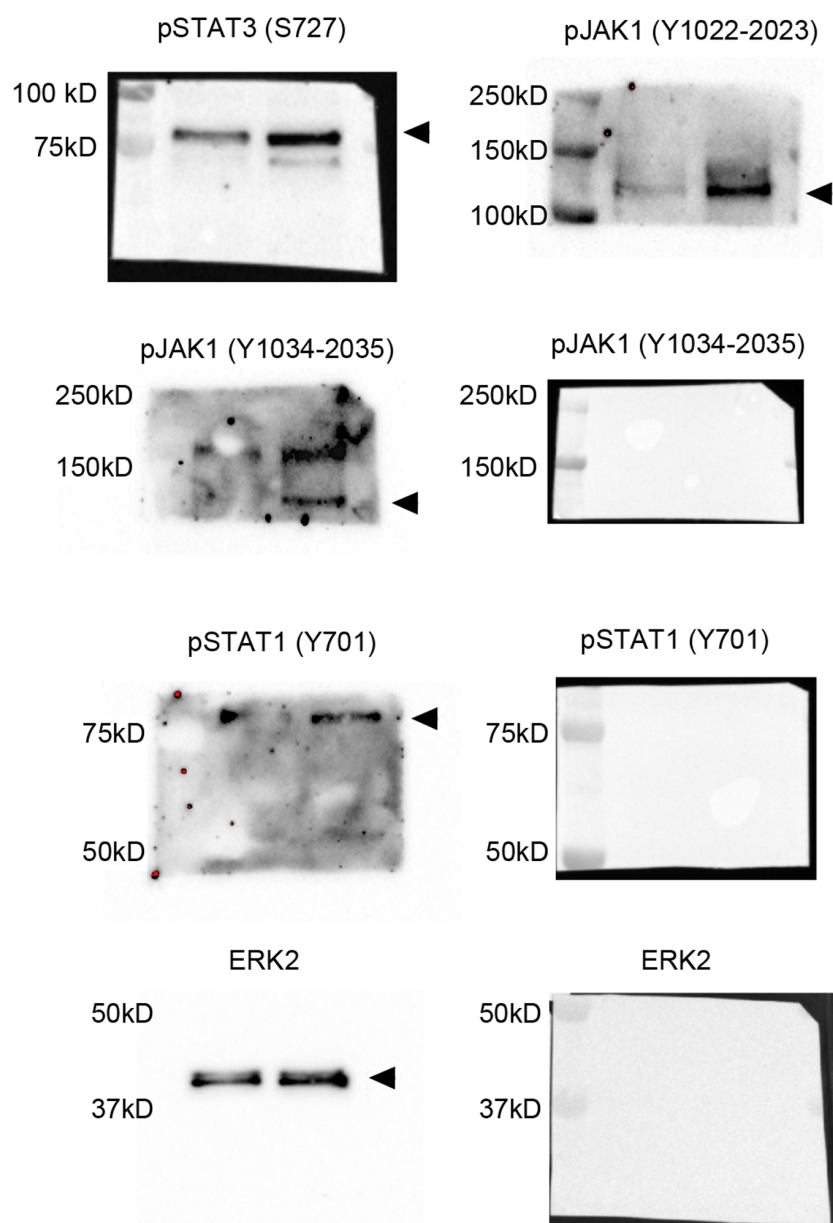

Supplement: Supplementary file 7 — Data from gene expression measurements and metabolomics. [file 42255_2023_861_MOESM7_ESM.pdf]
